# Oligomerization, trans-reduction, and instability of mutant NOTCH3 in inherited vascular dementia

**DOI:** 10.1038/s42003-022-03259-2

**Published:** 2022-04-07

**Authors:** Kelly Z. Young, Carolina Rojas Ramírez, Simon G. Keep, John R. Gatti, Soo Jung Lee, Xiaojie Zhang, Magdalena I. Ivanova, Brandon T. Ruotolo, Michael M. Wang

**Affiliations:** 1 Departments of Neurology, University of Michigan, Ann Arbor, MI 48109-5622 USA; 2 Molecular & Integrative Physiology, University of Michigan, Ann Arbor, MI 48109-5622 USA; 3 Department of Chemistry, University of Michigan, Ann Arbor, MI 48109 USA; 4 The Johns Hopkins School of Medicine, Baltimore, MD 21205 USA; 5 Biophysics Program, University of Michigan, Ann Arbor, MI 48105 USA; 6 Neurology Service, VA Ann Arbor Healthcare System, Ann Arbor, MI 48105 USA

**Keywords:** Protein aggregation, Stroke

## Abstract

Cerebral small vessel disease (SVD) is a prevalent disease of aging and a major contributor to stroke and dementia. The most commonly inherited SVD, CADASIL, is caused by dominantly acting cysteine-altering mutations in NOTCH3. These mutations change the number of cysteines from an even to an odd number, but the impact of these alterations on NOTCH3 protein structure remain unclear. Here, we prepared wildtype and four mutant recombinant NOTCH3 protein fragments to analyze the impact of CADASIL mutations on oligomerization, thiol status, and protein stability. Using gel electrophoresis, tandem MS/MS, and collision-induced unfolding, we find that NOTCH3 mutant proteins feature increased amounts of inappropriate disulfide bridges, reduced cysteines, and structural instability. Presence of a second protein factor, an N-terminal fragment of NOTCH3 (NTF), is capable of further altering disulfide statuses of both wildtype and mutant proteins, leading to increased numbers of reduced cysteines and further destabilization of NOTCH3 structure. In sum, these studies identify specific cysteine residues alterations and quaternary structure induced by CADASIL mutations in NOTCH3; further, we validate that reductive factors alter the structure and stability of this small vessel disease protein.

## Abstract (web summary)

Specific cysteine residue alterations and quaternary structures are induced by CADASIL mutations in NOTCH3, which are found to induce oligomeric states, altered disulphide bonding, increased free thiols and reduced protein stability.

## Introduction

Cerebral small vessel disease (SVD) is found in the majority of the aging population and is a powerful risk factor for stroke and dementia, including Alzheimer’s disease^1^. The best understood inherited SVD is cerebral autosomal dominant arteriopathy with subcortical infarcts and leukoencephalopathy (CADASIL), a condition marked by vascular smooth muscle cell degeneration, arterial thickening, and intimal hyperplasia^2^. CADASIL is known to result from over one hundred different mutations in the NOTCH3 gene, and the overwhelming majority of CADASIL-causing mutations change the number of cysteines from an even to an odd number^2^. The biochemical consequences of the CADASIL-causing mutations on NOTCH3 protein are unclear.

NOTCH3, like other Notch receptors and their ligands, features 34 tandemly arranged Epidermal Growth Factor (EGF)-like repeats in the ectodomain region, where the vast majority of CADASIL causing mutations are found^3^. Each EGF-like repeat features six evolutionarily conserved cysteines, and solved structures of EGF-like domains demonstrate that the cysteines form three intradomain disulfide bonds^3,4^. As such, an odd number of cysteines may interfere with normal disulfide pairing. Resulting abnormalities could include the formation of electrochemically reduced thiols that remain unpaired with other cysteines or of mispaired disulfides (both inter- and intramolecular pairs). Mispaired disulfides may cause aberrant protein oligomerization and misfolded or unstable proteins^5^. While mutant NOTCH3 proteins differ from wildtype (WT) protein in glycosylation^6^ and multimerization^5^, the role of cysteines in these processes has not been explored^5–7^.

The presence of disulfide abnormalities in a population of NOTCH3 protein has been inferred in prior studies, which identified a form of NOTCH3 in diseased human vessels that appear conformationally similar to NOTCH3 resulting from multiple reduced cysteines^8^. Although several studies utilizing 3-D modeling predict that a variety of CADASIL causing mutations considerably affect protein structure^3,4^, experimental evidence of excessive thiols, mispaired disulfide bonds, and structural lability of mutant proteins is limited.

The pathology of CADASIL reveals massive protein accumulation that includes NOTCH3 protein. Little evidence is available about whether mutant NOTCH3 is more stable and resistant to clearance, resulting in toxic protein build. Alternatively, mutant NOTCH3 proteins could also be more labile, enabling structural alterations that unmask reactive chemical side chains (such as thiol groups), resulting in the promotion of disease by molecules that react with proteins essential for cell homeostasis. A first step to differentiating between molecular buildup or molecular lability requires an assessment of the impact of disease-linked mutations on NOTCH3 stability. In this study, we characterized the thiol states of WT and four NOTCH3 mutants and compared their stabilities in vitro. We further evaluate the capability of a fragment of NOTCH3 to affect NOTCH3 disulfide status and stability.

## Results

### CADASIL mutant NOTCH3 forms more higher-order multimers than WT NOTCH3

The large size of NOTCH3 prevents facile study of the full protein, but the modular nature of the ectodomain has enabled the study of soluble fragments. We focus on the first three EGF-like repeats of NOTCH3, where some of the most common and best characterized disease-causing mutations occur, as a model to shed light on disease-related changes. Initial studies were conducted to determine differences in gel mobility between WT and mutants.

**Fig. 1:**
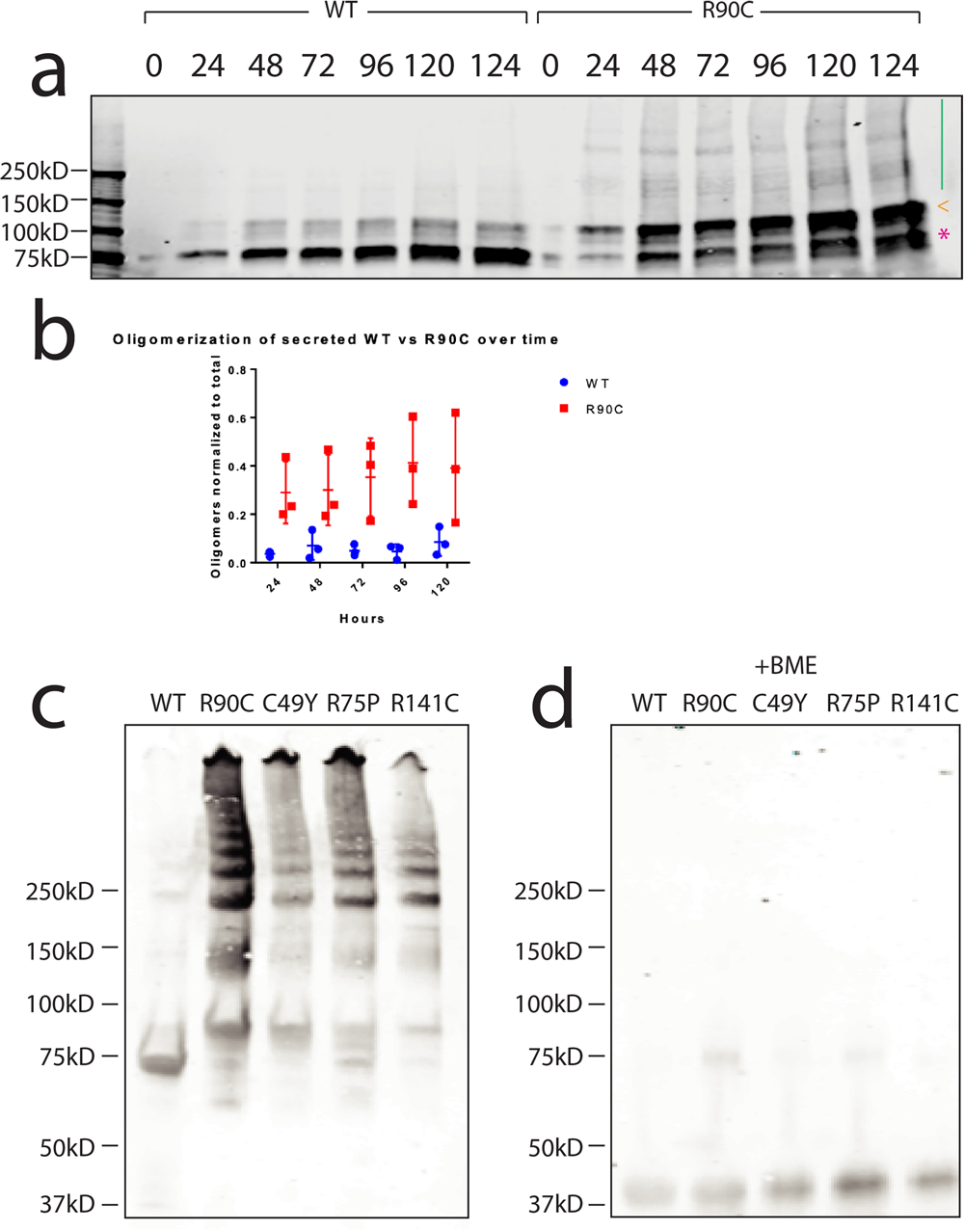
Mutant NOTCH3 forms larger multimers compared to WT NOTCH3. On nonreducing SDS-PAGE gels, we observed higher-order multimerization (green line) of the R90C protein at various time points compared to WT protein, which only formed Fc dimers (pink * and orange <) (**a**). Comparison of R90C and WT multimer protein content to total Fc-NOTCH3 protein content from the media demonstrated the increased presence of R90C multimers in the media (**b**). R90C multimer content did not change with increased hours post-transfection (**b**). Center line represents the mean; upper and lower lines designate the standard deviation. Recombinant WT and mutant NOTCH3 protein were purified from stably transfected cell lines, as previously described^9^. All proteins were examined on nonreducing 4–20% SDS-PAGE gels (ThermoFisher). Protein laddering was observed for all mutants examined: R90C mutant, C49Y mutant, R75P mutant, and R141C mutant (**c**). Little to no laddering was observed for WT protein (**c**). The addition of ß-mercaptoethanol completely reduced multimers to the monomer level (**d**). Experiments were performed at least three times on distinct samples with similar results. Unprocessed gels can be found in Fig. S8.

Recombinant WT or mutant NOTCH3 ectodomain fragments containing the first three EGF-like domains fused to Fc were prepared by transfection^9^. WT and R90C proteins secreted to the media were examined by western blotting. On reducing SDS-PAGE gels, WT and R90C mutant NOTCH3 share a single band of indistinguishable mass. However, in the absence of reducing agents, secreted R90C protein had a major band that co-migrated with non-reduced WT protein but also displayed higher molecular weight species in a laddering pattern indicating multimer formation larger than dimers (multimers labeled by green line; dimers are expected for Fc fusion proteins; Fig. 1a). A time course of incubation of these proteins demonstrated that multimerization of R90C is present at 24 h, and multimer formation remains relatively stable with increased lengths of incubation (Fig. 1b).

To investigate whether multimer formation is a more general property of CADASIL mutants, we then compared WT and four CADASIL-mutant proteins, which were purified from cell lines stably expressing Fc-fused NOTCH3 ectodomains by protein A affinity chromatography^9^. In addition to cysteine altering NOTCH3 mutants R90C, C49Y, and R141C, we analyzed a non-cysteine mutant R75P in order to address whether cysteine-involving and non-cysteine involving mutations exhibit convergent properties. Examination of fresh protein on non-reducing gels revealed a laddering pattern for all four mutants tested, with no detectable laddering above the dimer bands shared with the WT protein (Fig. 1c). On the other hand, the addition of a reducing agent before electrophoresis (which served as a control for the amount of protein loaded) virtually eliminated all mutant multimers (Fig. 1d).

### Mutant NOTCH3 harbors increased amounts of free thiols compared to WT

The increased propensity for mutant NOTCH3 to form disulfide-dependent intermolecular complexes indicated that these proteins may also contain increased amounts of unpaired thiols capable of forming bridging bonds. To quantify the burden of unpaired thiols in WT versus mutant NOTCH3, we used differential cysteine labeling followed by tandem mass spectrometry (MS/MS) of the previously described purified Fc-tagged WT and four mutant NOTCH3 protein fragments^10^. To identify native free thiols in WT and mutant NOTCH3, N-ethylmaleimide (NEM) was added in excess to the purified protein. We then reduced the NEM-labeled protein to liberate thiols that had not previously reacted with NEM. Newly freed thiols were immediately labeled with an excess of 2-chloroacetamide, trypsinized, and examined with MS/MS.

Peptides were identified for 10 of the 12 predicted tryptic fragments, and all cysteines of interest were visualized. Proportions of NEM-labeled cysteines were calculated from NOTCH3 peptide spectrum matches (PSMs). The vast majority of cysteines in WT NOTCH3 were labeled with 2-chloroacetamide, with few free thiols (0.93%) available for NEM labeling. On the other hand, all mutants examined (R90C, R141C, C49Y, and R75P) had increased proportions of cysteines labeled with NEM (2.01%, 4.98% *p* < 0.001, 1.94%, and 2.10%, respectively; Fig. S1a).

**Fig. 2:**
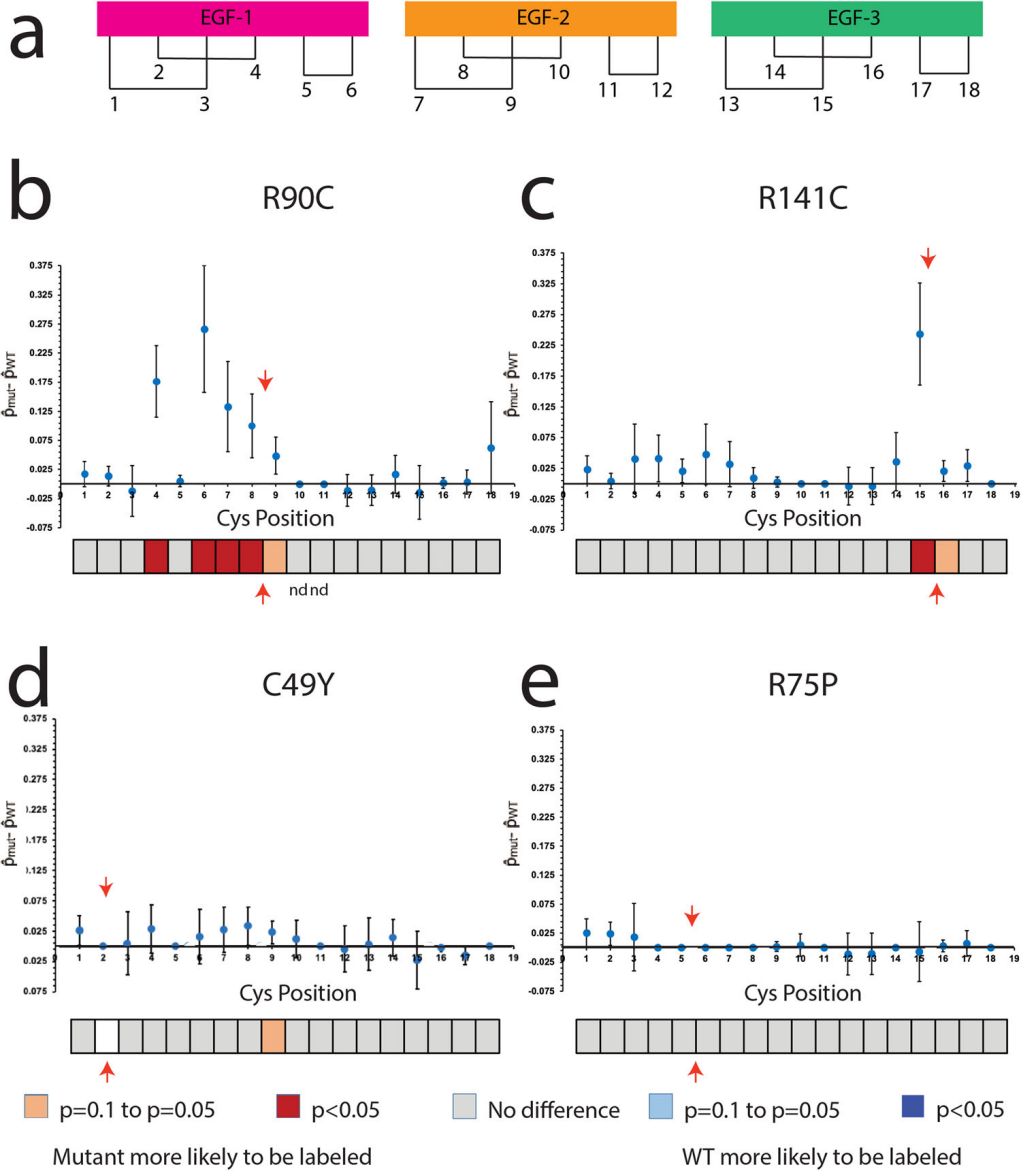
Select CADASIL mutant proteins have increased probability of having free thiols accessible for NEM labeling compared to WT protein. **a** Demonstrates a schematic of the first 3 EGF like repeats with the predicted disulfide pairing. All cysteines examined are labeled from 1 to 18. Cysteines that are only present in mutant proteins are not shown but can be found in Fig. S1. R90C mutant protein demonstrated a significantly increased probability of being labeled with NEM at cysteine positions 4, 6, 7, and 8 (*p* < 0.05; (**b**)). The R141C mutant protein had significantly increased probabilities of being labeled with NEM at position 15 (*p* < 0.05) and trended toward increased NEM labeling at position 16 (**c**). The C49Y mutant did not significantly differ in NEM labeling probabilities from WT protein, but the mutant protein trended towards having an increased probability of NEM labeling at cysteine position 9 (**d**). The R75P mutant protein did not differ significantly from WT protein in NEM labeling at any cysteine position (**e**). The bar below each panel represents statistical significance at each cysteine position (box). Locations of each mutation found in the mutant proteins are indicated with a red arrow. Cysteines that were not detected in a sample are denoted with “nd.” Experiments were repeated at least six times using distinct samples with similar results. Figure 2 includes data from all replicates performed. Individual data points used for analysis can be found in Fig. S1b–e.

Further analysis of cysteine labeling relative to cysteine position within the NOTCH3 fragment was conducted. Cysteines were identified and numbered from positions 1 to 18 (Fig. 2a), and the proportion of NEM labels at each position was calculated from the total number of cysteines observed at that position (PSMs).

We found that 95.24% of R90C residues were unavailable to NEM in the R90C mutant and 93.17% of R141C residues were unavailable to NEM in the R141C mutant (Fig. S1b–c). In addition, we identified cysteines outside of the expected mutations that demonstrated increased NEM labeling (Fig. S1b–e). To test whether the probability of NEM labeling at a specific cysteine position is significantly different between WT and mutant NOTCH3, we calculated the test statistic (see methods) and found significantly increased probabilities of obtaining an NEM label at cysteine positions 4, 6, 7, and 8 in the R90C mutant compared to WT protein (*p* < 0.05; red boxes in Fig. 2b). In addition, we found a significantly increased probability of obtaining an NEM label at cysteine position 15 in the R141C mutant compared to WT protein (*p* < 0.05; Fig. 2c). Interestingly, these regions clustered near the location of each mutation (Fig. 2b–c, red arrows). In contrast, the probabilities of obtaining an NEM label at any of the cysteines in the C49Y mutant and R75P mutant were not statistically different from those of WT NOTCH3, although a positive trend was observed at position 9 in the C49Y mutant (Fig. 2d–e).

The presence of unpaired cysteines with positional preference increases the likelihood of mutant fragments containing multiple reduced cysteines in a single molecule of NOTCH3. We identified occasional single tryptic fragments with >1 NEM label, demonstrating the existence of NOTCH3 populations with multiple reduced cysteines (Fig. S2a). Tryptic fragments harboring multiple NEM labels were identified in all mutant and WT proteins, while the R90C and R141C proteins trended towards having increased detection of tryptic fragments with >1 NEM label (Fig. S2b, ns).

### Interaction of NOTCH3 with an N-terminal fragment of NOTCH3 (NTF)

Previous work suggested that in CADASIL arteries, mutant NOTCH3 is present in extensively reduced states^8^. Although we found reduced thiols in some mutants and evidence for multiple reduced cysteines, these forms were in the minority in purified protein preparations. We, therefore, sought to determine if mutant NOTCH3 could be preferentially reduced by factors present in disease vessels that were not present in purified protein preparations. In the degenerating media of CADASIL vessels, NOTCH3 is cleaved at an aspartate-proline bond at the N-terminus^11^. The predicted product of the cleavage is a 41 amino acid fragment of NOTCH3, N-terminal fragment (NTF). Synthetic NTF has been shown to spontaneously multimerize, drawing parallels to the oligomerizing properties demonstrated by other proteins found in neurodegenerative diseases^11^. Given that both NTF and full-length NOTCH3 are found in the degenerating media of diseased vessels, we performed a series of studies to determine if the addition of NTF could alter the thiol content of mutant NOTCH3 proteins.

**Fig. 3:**
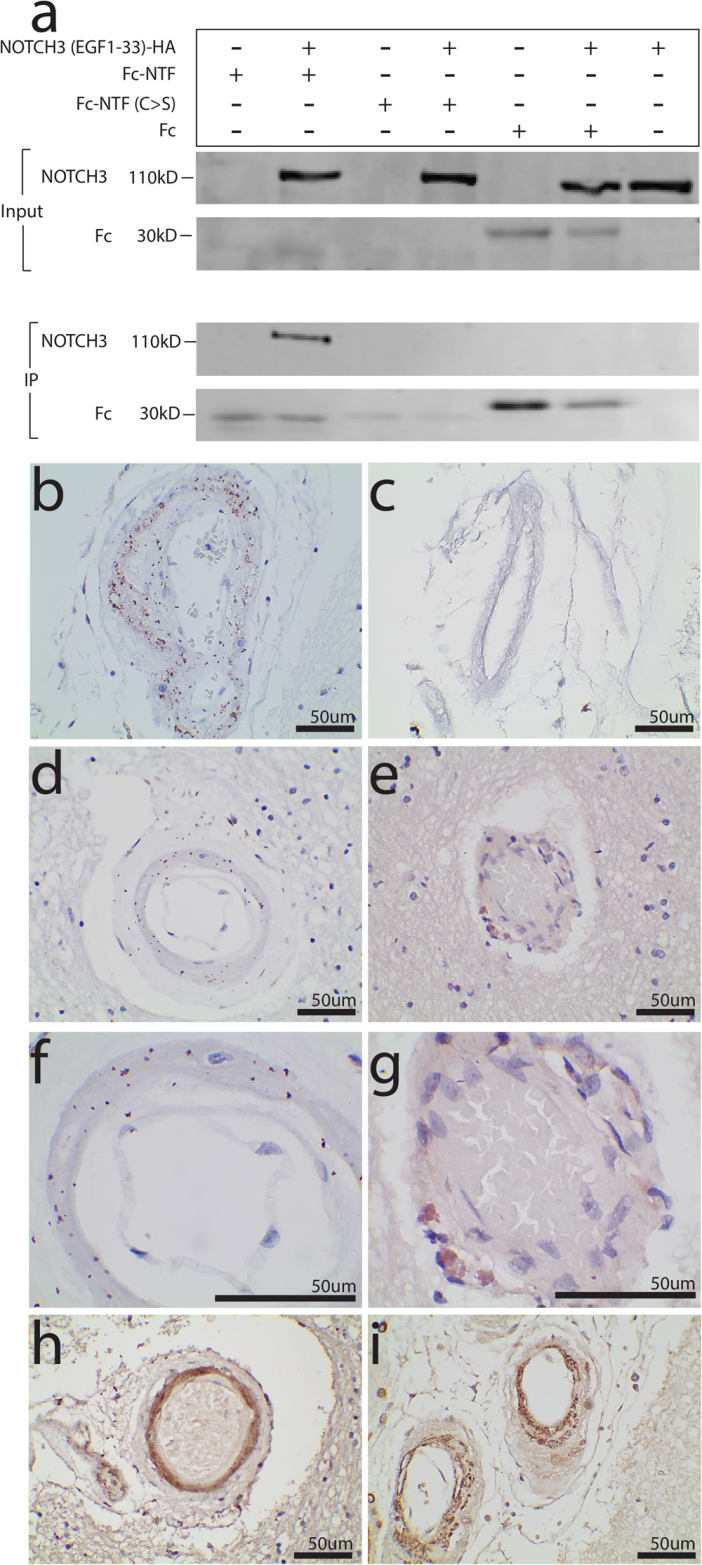
NTF forms complexes with NOTCH3 detectable in CADASIL vessels. Fc-NTF co-immunoprecipitated with NOTCH3-HA (**a**). A version of Fc-NTF with all cysteines mutated to serine (Fc-NTF C > S) and the Fc tag alone were unable to pull down NOTCH3-HA. Experiments were performed at least three times using distinct samples with similar results. Unprocessed western blots can be found in Fig. S9. PLA was performed on paraffin-embedded postmortem CADASIL and control brains using an antibody specific for NTF (UMI-F^11^) and a second antibody recognizing a different epitope of NOTCH3 (1E4; Sigma Aldrich^30^). Positive PLA signal was identified in 4/4 CADASIL brains and 0/3 age-matched control brains. A representative leptomeningeal vessel from a CADASIL brain is displayed in (**b**) at ×400 and a representative leptomeningeal vessel from a control is displayed in (**c**) at ×400. A representative penetrating white matter vessel from a CADASIL brain is displayed in (**d**) at ×400 magnification and in (**f**) at ×1000 magnification, and a representative penetrating white matter vessel from a control vessel is displayed in (**e**) at ×400 magnification and in (**g**) at ×1000 magnification. Representative vessels from a CADASIL brain stained with UMI-F and 1E4 by immunohistochemistry are shown at ×400 in (**h**) and (**i**), respectively.

First, we tested if NTF can form complexes with NOTCH3 in vitro by conducting immunoprecipitation studies with an Fc-tagged fragment of NTF (Fc-NTF) and an HA-tagged fragment of NOTCH3 containing 33 EGF-like repeats (NOTCH3-HA). We observed that Fc-NTF could pull down NOTCH3-HA, while a version of Fc-NTF with all cysteines mutated to serines (Fc-NTF C > S) was unable to pull down NOTCH3-HA (Fig. 3a).

Second, we tested whether NTF-NOTCH3 interaction could occur in vivo. To do so, we used proximity ligation assay (PLA) with an antibody specific for NTF (UMI-F, which has been shown to be specific for the epitope revealed by cleavage of NOTCH at Asp41^11^) and an antibody that recognizes an epitope near the middle of the NOTCH3 ectodomain (1E4, EMD Millipore). Positive PLA signal is observed when the two proteins of interest are within 40 nm apart, supporting protein-protein interaction. We found positive PLA signal in 4/4 CADASIL brains (Fig. 3b, d, f) and 0/3 age-matched control brains (Fig. 3c, e, g). PLA signal localization is consistent with medial protein staining and overlaps with regions stained with UMI-F (Fig. 3h) and 1E4 (Fig. 3i).

### Liberation of NOTCH3 thiol groups by an N-terminal fragment of NOTCH3 (NTF)

**Fig. 4:**
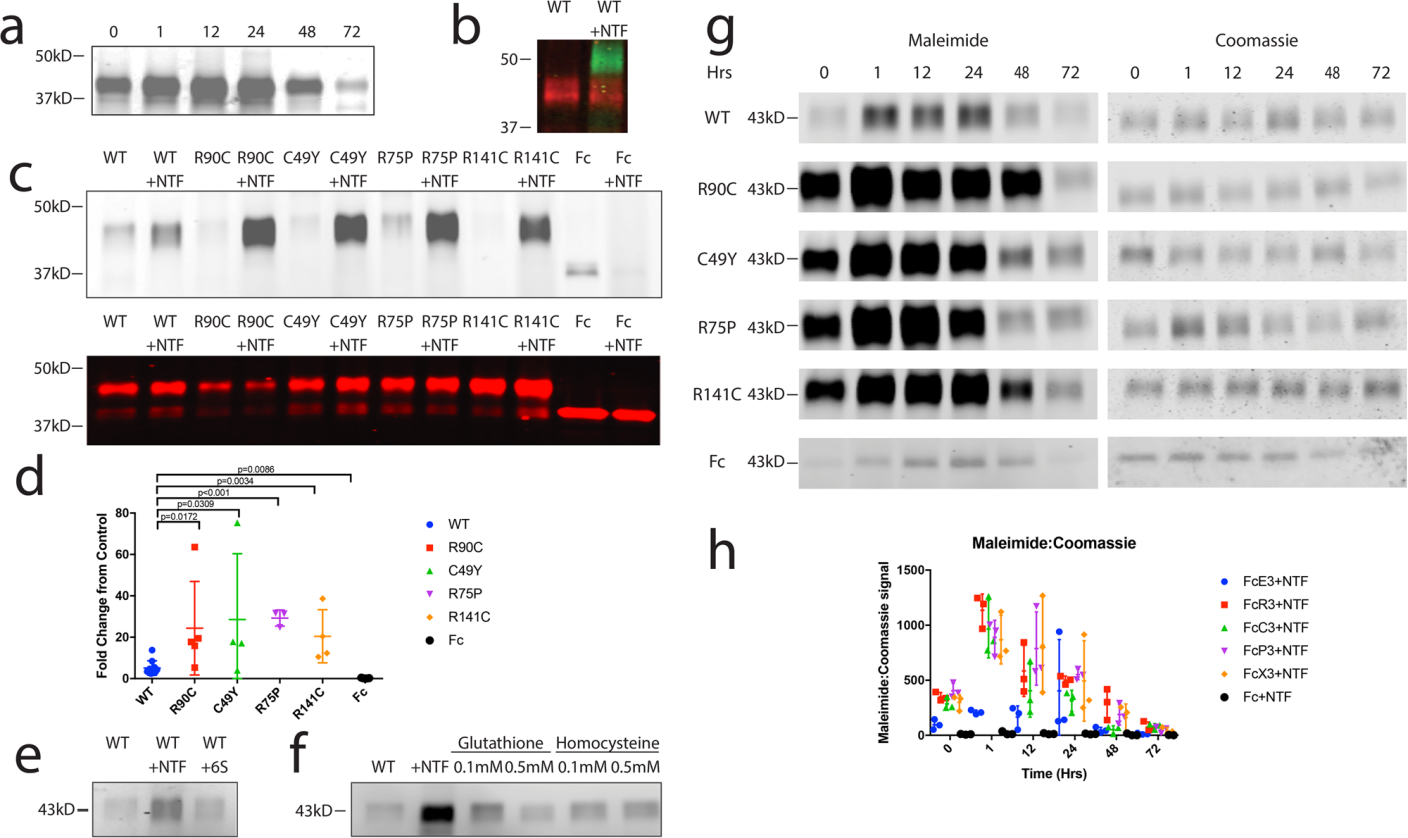
NTF releases cysteines in NOTCH3 and makes them accessible for thiol-labeling. Following the addition of NTF to Fc-tagged NOTCH3 containing the first 3 EGF-like repeats, we observed increased maleimide signal with increased lengths of incubation with NTF until 24 h, after which signal decreased (**a**). Detection of NTF using an antibody specific for the cleaved fragment of NOTCH3, UMI-D, revealed a complex at a size larger than the NOTCH3 alone size (**b**; green). Purified NOTCH3 was identified by probing for the Fc tag (**b**; red). We observed increased maleimide signal with the addition of NTF to all WT and mutant samples, while the addition of NTF to Fc protein alone did not increase signal (**c**, upper panel). A parallel set of samples was transferred to nitrocellulose using the iBlot 2 system and protein loading amounts were verified by probing for Fc (**c**, lower panel). Quantification of maleimide signal in samples with and without NTF demonstrated 5.13-fold enhancement in WT protein and 24.37-fold, 28.55-fold, 29.26-fold, and 20.44-fold enhancement in mutant proteins R90C, C49Y, R75P, and R141C, respectively (**d**). The addition of NTF to Fc resulted in 20% signal compared to without NTF (**d**). Effects of NTF on purified WT NOTCH3 were compared to those from a form of NTF with all cysteines mutated to serine (6S). The addition of NTF to NOTCH3 resulted in an increased maleimide signal, while no increase was observed with the addition of 6S to NTF (**e**). Similarly, no increase was observed with the addition of two concentrations (0.1 and 0.5 mM) of two other sulfhydryl-containing compounds, glutathione and homocysteine (**f**). NTF was then added to purified Fc-tagged NOTCH ectodomain fragments for increasing lengths of time. Cysteines were then capped with 10 μM IRDye 800CW Maleimide (**g**, left panel). Total protein content was determined by SimplyBlue Safestain (ThermoFisher) (**g**, right panel). Quantification of maleimide signal normalized to total protein amount revealed increasing maleimide signal that peaks at 1 h for all mutants and decreases with longer incubation times (**h**). WT protein signal peaks around 24 h. Experiments were repeated three times using distinct samples with similar results, and (**h**) plots the mean of three replicates. Unprocessed western blots can be found in Fig. S10.

Having established that NTF can bind to NOTCH3 in vitro and is present in close association with NOTCH3 in vivo, we performed studies to investigate whether NTF could alter the disulfide bonds and thiol content of NOTCH3. Due to the importance of cysteines in the interaction between NTF and NOTCH3 (Fig. 3a), we tested whether NTF could affect NOTCH3 disulfide bonds. We incubated purified recombinant Fc-NOTCH3 containing the first three EGF-like repeats with and without synthetic NTF^12^ at 1:50 molar ratio for increasing lengths of time at 37 °C. We then labeled free thiols with a fluorescent maleimide (IRDye 800CW Maleimide, Licor), and separated the Fc-NOTCH3 and NTF bands on reducing SDS-PAGE gels (Fig. 4a). With the addition of NTF to NOTCH3, we observed a time-dependent increase in maleimide cysteine labeling of proteins migrating at the expected molecular weight of the WT NOTCH3 fragment (Fig. 4a).

Since NTF can bind to NOTCH3 in vitro (Fig. 3a), we examined whether the increase in maleimide signal could be attributed to NOTCH3-bound NTF. We probed NTF treated NOTCH3 samples with an antibody specific for the cleaved NOTCH3 product, NTF (UMI-D which is specific for the epitope created by NOTCH3 cleavage^11^). When doing so, we identified a UMI-D reactive band above the expected weight for purified recombinant NOTCH3 (Fig. 4b green), demonstrating that the NTF-NOTCH3 complex that does not comigrate with NOTCH3 (Fig. 4b red).

We then compared the ability of NTF to reduce Fc-tagged mutant and WT NOTCH3. The addition of NTF to NOTCH3 consistently increased maleimide signal of both WT and mutant NOTCH3 proteins (Fig. 4c top panel), while protein loading amounts were similar (Fig. 4c bottom panel). The addition of NTF to Fc protein alone did not increase maleimide signal in Fc (Fig. 4c). Quantification of maleimide signal as fold change in signal of samples with NTF over the signal of samples without NTF demonstrates that NTF addition to WT protein results in a 5.13 fold increase in signal compared to WT protein alone, while NTF addition to mutants R90C, C49Y, R75P, and R141C resulted in 24.37 fold, 28.55 fold, 29.26 fold, and 20.44 fold increases compared to mutant protein alone, respectively (Fig. 4d). Mutation of all cysteines to serine in NTF (6S) eliminated the ability of the peptide to trans-reduce NOTCH3 protein (Fig. 4e). Finally, we investigated whether the increase in free thiols of NOTCH3 with the addition of NTF was specific to NTF or a general effect of introducing an excess of sulfhydryl-containing compounds. We compared the addition of NTF to two concentrations of sulfhydryl-containing compounds glutathione and homocysteine. 0.1 mM glutathione and 0.1 mM homocysteine are roughly equimolar to the amount of NTF added in prior experiments and 0.5 mM glutathione and 0.5 mM homocysteine are roughly equimolar to the number of cysteines in NTF. We found that neither addition of glutathione nor homocysteine at these concentrations was able to reduce NOTCH3 (Fig. 4f).

The ability of NTF to reduce NOTCH3 was exceedingly fast, with a fraction of the protein demonstrating large amounts of available thiols immediately upon addition (Fig. 4g Hr 0), which was most apparent with mutant proteins (Fig. 4g–h). The number of free thiols in most proteins peaked at 1 h after the addition of NTF and decreased over time, suggesting reoxidation (Fig. 4h).

Furthermore, NTF addition to Fc-NOTCH3 visualized on non-reducing gels (Fig. S3a) compared to reducing gels (Fig. S3b) altered the laddering pattern of Fc-NOTCH3 proteins. Quantification of protein laddering normalized to monomer amount revealed a trend towards increased proportions of multimeric species with the addition of NTF in all proteins tested (Fig. S3c). However, only the proportion of laddering of WT protein was significantly altered by NTF (Fig. S3c; *p* = 0.02).

**Fig. 5:**
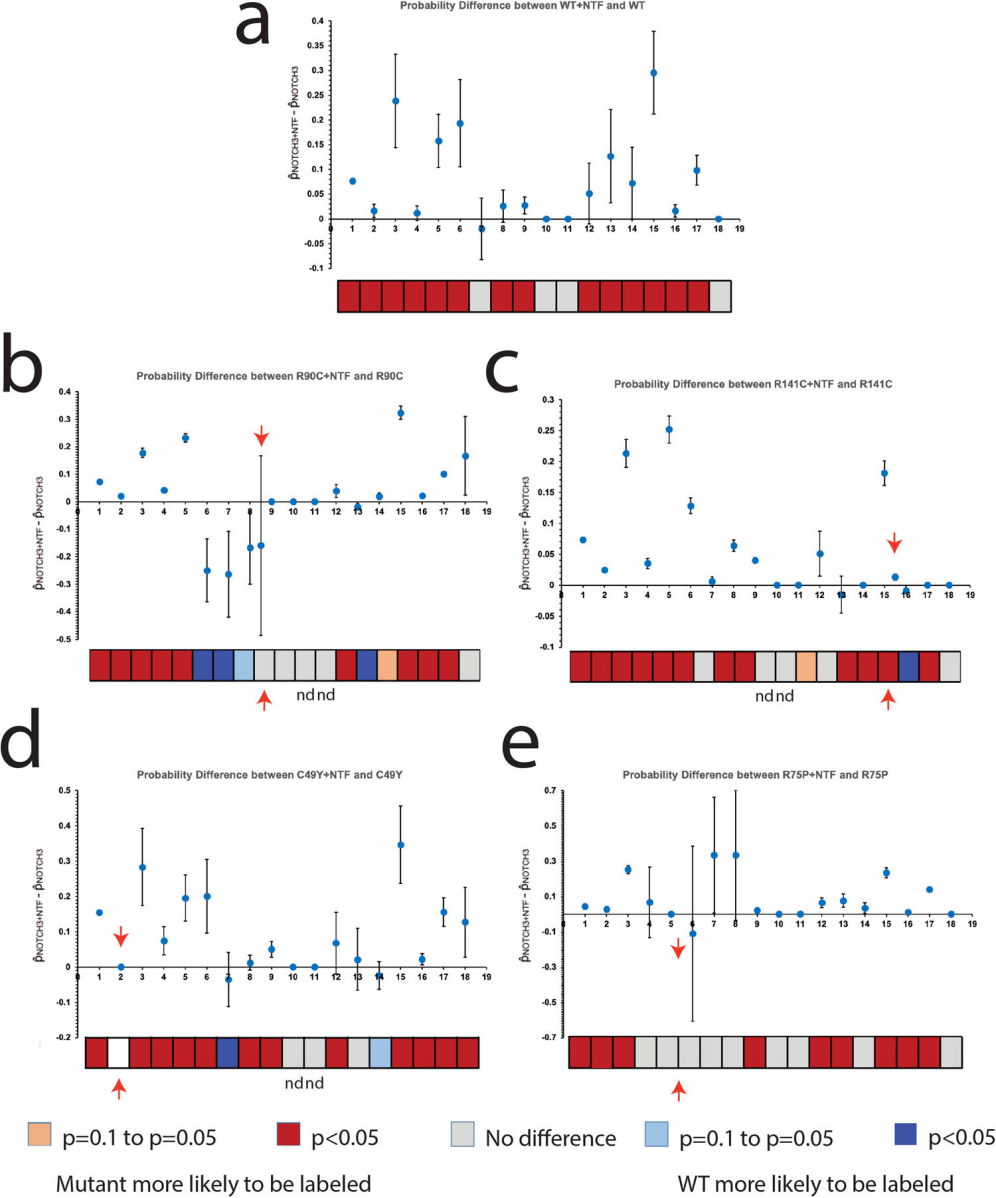
Thiols liberated by NTF are detected by MS/MS. NTF was added to purified Fc-tagged fragments of NOTCH3 and examined with MS/MS. The probabilities of each cysteine being labeled with NEM were calculated and compared between the samples with NTF and samples without NTF. The addition of NTF to WT NOTCH3 significantly increased the probabilities of a cysteine being labeled with NEM at positions 1, 2, 3, 4, 5, 6, 8, 9, 12, 13, 14, 15, 16, and 17 (**a**). The addition of NTF to R90C mutant protein significantly increased probabilities of cysteines 1, 2, 3, 4, 5, 12, 15, 16, and 17 being labeled with NEM and significantly decreased probabilities of cysteines 6, 7, and 13 being labeled with NEM (**b**). The addition of NTF to R141C mutant protein significantly increased probabilities of cysteines 1, 2, 3, 4, 5, 6, 8, 9, 14, 15, 17, and R141C being labeled with NEM and significantly decreased probability of cysteines 16 being labeled with NEM (**c**). The addition of NTF to C49Y mutant significantly increased probabilities of getting an NEM label at cysteine positions 1, 3, 4, 5, 6, 8, 9, 12, 15, 16, 17, and 18 and significantly decreased probabilities of getting an NEM label at position 7 (**d**). Finally, the addition of NTF to R75P mutant protein significantly increased probabilities of observing an NEM label at cysteine positions 1, 2, 3, 8, 12, 13, 15, 16, and 17 (**e**). The bar below each panel represents statistical significance at each cysteine position (box). All locations of mutations are marked with a red arrow, and cysteines that were not detected were labeled with “nd.” Each experiment was repeated three times using distinct samples, and the results from all three replicates are displayed in Fig. 5. Error bars represent the standard deviation. Individual data points used for analysis can be found in Fig. S4c–g.

To map the specific regions of NOTCH3 protein that were susceptible to reduction by NTF, we utilized differential cysteine labeling and MS/MS to observe thiol statuses at the single cysteine level, as described above. In brief, we compared the labeling of free cysteines (NEM) in WT and mutant proteins with and without the addition of NTF. Prior to MS/MS, proteins were separated by reducing SDS-PAGE, and only bands at the size of purified Fc-tagged NOTCH3 were examined. In the analysis, we confirmed the absence of NTF-specific tryptic peptides. Overall, the addition of NTF increased the proportion of cysteines labeled with NEM in both WT and mutant proteins using two methods of NEM label detection: immunodetection of NEM labeled proteins (Fig. S4a, Absolute Antibody^13^) and MS/MS (Fig. S4b). NTF addition to NOTCH3 resulted in the liberation of the vast majority of cysteines for reaction with NEM (Fig. S4C–G; Fig. 5a–e). There were no obvious regional differences between WT and mutant protein and between the four different mutant proteins examined (Fig. S5).

### Stability of non-oligomeric mutant NOTCH3 ectodomain in vitro

We employed two approaches to compare the stability of WT and mutant NOTCH3: (1) ion mobility-mass spectrometry (IM-MS) to identify the spectrum of masses of non-oligomeric protein and (2) collision-induced unfolding (CIU) to further distinguish protein isoforms by their unfolding pathways^14,15^.

**Fig. 6:**
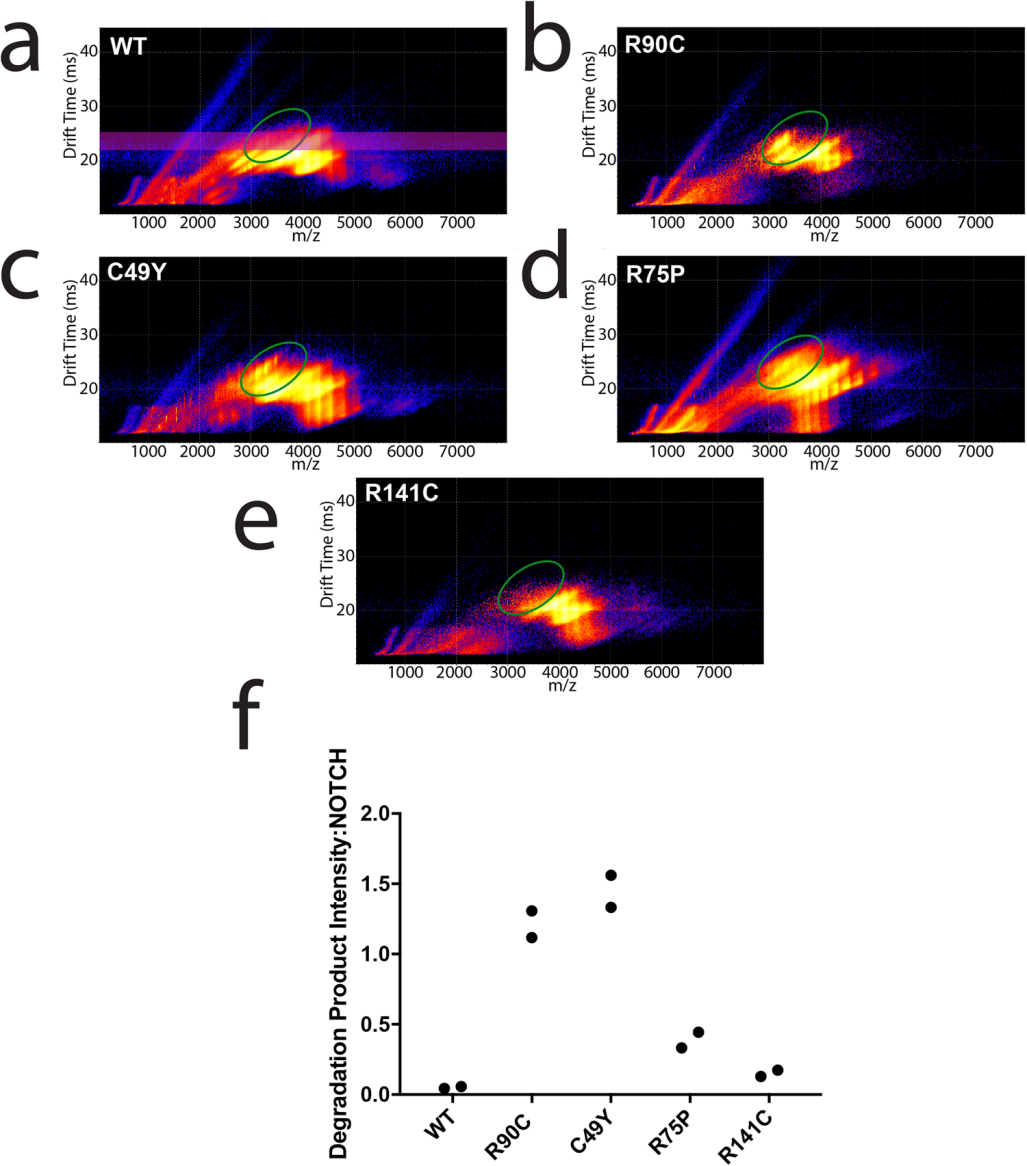
IM-MS identifies a population of degradation products in selected mutants. IM-MS plots from two experimental replicates demonstrated the presence of an entity appearing at 100 V for R90C, C49Y, and R75P mutant proteins (**a**–**e**, green circle). Investigation of WT and R141C protein failed to qualitatively identify this population. Further analysis of the C49Y mutant protein revealed an approximate mass of 55 kDa, smaller than the theoretical 86 kDa size of the purified Fc-fusion proteins. This degradation product was absent at lower voltages for all constructs (Fig. S6). Quantification of degradation product intensity relative to intact construct calculations is displayed in (**f**). By extracting the IM region of 22–25 (**a**, pink bar) both the degradation product and intact NOTCH3 intensities were extracted. Increased amounts of degradation product were observed in the R90C, C49Y, R75P, and R141C mutants (**f**). The ratio of degradation product to intact NOTCH3 was lowest for WT protein.

Using IM-MS, all Fc-fusion protein constructs had an approximate mass of 86 kDa, as expected from theoretical sequences. Molecular profiling at 100 V excitation revealed that mutants R90C, C49Y, and R75P and R141C generated increased amounts of a fragmentation product of ~55 kDa, compared to WT protein (Fig. 6a–e, green circle). This fragmentation product was not present at lower voltages (Fig. S6a–e). Quantification of the fragmentation product signal intensity normalized to the NOTCH3 protein within a specified drift time window (22–25 ms) is displayed in Fig. 6f. R90C, C49Y, R75P, and R141C mutants demonstrated increased amounts of fragmentation product compared to WT protein (Fig. 6f).

Since the resolution of our IM-MS platform is limited and is unable to distinguish subtly different structural differences, we employed CIU, an approach best viewed as a gas-phase analog of differential scanning calorimetry, to provide further insight into the stabilities of NOTCH variants^14^. By plotting the collision energy (V) required to unfold the protein against ion mobility in drift time (ms), we generated CIU fingerprint plots that can be compared in order to distinguish features of protein structures. Representative CIU fingerprints for WT and mutant proteins are displayed in Fig. S7.

**Fig. 7:**
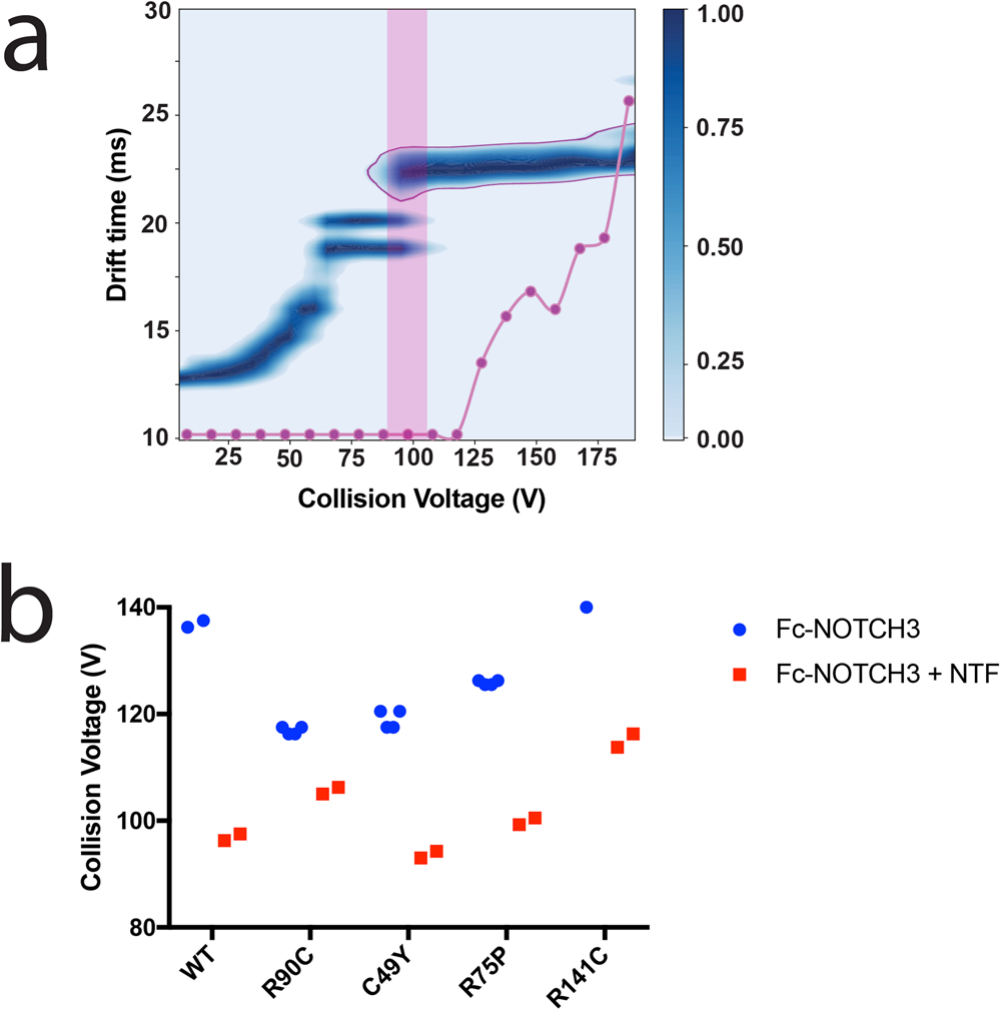
Stability Comparisons for Fc-Notch3 constructs with and without NTF-binding. **a** Demonstrates a collision-induced unfolding fingerprint of Fc-NOTCH3 WT protein bound to NTF. In all CIU fingerprints of Fc-constructs, four main features were observed. We selected the 22.5 ms feature for stability comparison (**a**, outlined in purple). NTF addition resulted in NTF-NOTCH3 binding identifiable by CIU (**a**). Dissociation of the NTF-NOTCH3 complex is tracked by the purple line in (**a**) and revealed increased amounts of unbound NOTCH3 protein at the feature traveling at a drift time of 22.5 ms (outlined in purple). Thus, we selected this final transition for comparison between WT and mutant proteins (**a**, pink box). CIU fingerprints for all proteins are displayed in Fig S7. Comparison of the Fc-Notch3 construct stability shifts (**a**, pink box) are displayed in (**b**). WT (*n* = 2) and R141C proteins (*n* = 2) required the highest onset voltages for the 22.5 ms feature (**b**). R90C (*n* = 4), C49Y (*n* = 4), and R75P mutants (*n* = 4) have a lower voltage requirement for the transition (**b**, orange asterisks). The addition of NTF resulted in a decrease in the onset voltage for the 22.5 ms feature in all constructs (**b**).

Overall, WT and mutant NOTCH3 protein undergo two major unfolding events that give rise to four shared features (Fig. 7a), and each feature corresponds to conformational states of the protein ion. All proteins tested had at least two distinct conformations of NOTCH3 proteins, as demonstrated by two populations of proteins in feature 2 with different drift times (Fig. 7a, Fig. S7). As we excited the proteins with higher voltages, more extended forms, indicated by larger drift times, are accessed. Comparing WT (Fig. S7a) and mutant fingerprints (Fig. S7b–e), we observed that less collisional voltage was required to unfold the R90C, C49Y, and R75P mutant proteins tested at the specified transition point (Fig. 7a, pink region; Fig. 7b). R141C did not differ in stability compared to WT protein (Fig. 7b).

### Addition of NTF destabilizes both WT and mutant NOTCH3

As seen above, NTF drastically increases the number of reduced thiols in NOTCH3, which is expected to alter its stability. To examine how NTF addition affects protein structure and stability, we acquired CIU fingerprints of purified WT and mutant NOTCH3 protein fragments with and without the addition of NTF at a 1:25 molar ratio for 1 h at 37 °C. We found that NTF binds to both WT and mutant NOTCH3 fragments and were able to visualize the dissolution of the NTF-NOTCH3 complex with the addition of increased energy (at voltages ≥ 100 V; Fig. 7a). The addition of NTF further destabilized WT and mutant proteins, requiring less energy to unfold to the final conformation (Fig. 7b; Fig. S7).

## Discussion

The molecular genetics of CADASIL have strongly implicated a pathogenic role for NOTCH3 cysteine abnormalities, and the work presented here contributes several insights into how thiol and disulfide cysteines affect the NOTCH3 protein. The key findings include: (1) four independent mutant NOTCH3 proteins assemble into multimers whose formation requires disulfide bonds; (2) selected mutants harbor abnormal disulfide bonds and increased free thiols across EGF-like domains; (3) selected mutants demonstrate increased spontaneous instability compared to WT protein; (4) all four mutant NOTCH3 are preferentially sensitized to reduction by NTF; (5) selected mutants and all trans-reduced NOTCH3 are inherently unstable.

**Fig. 8:**
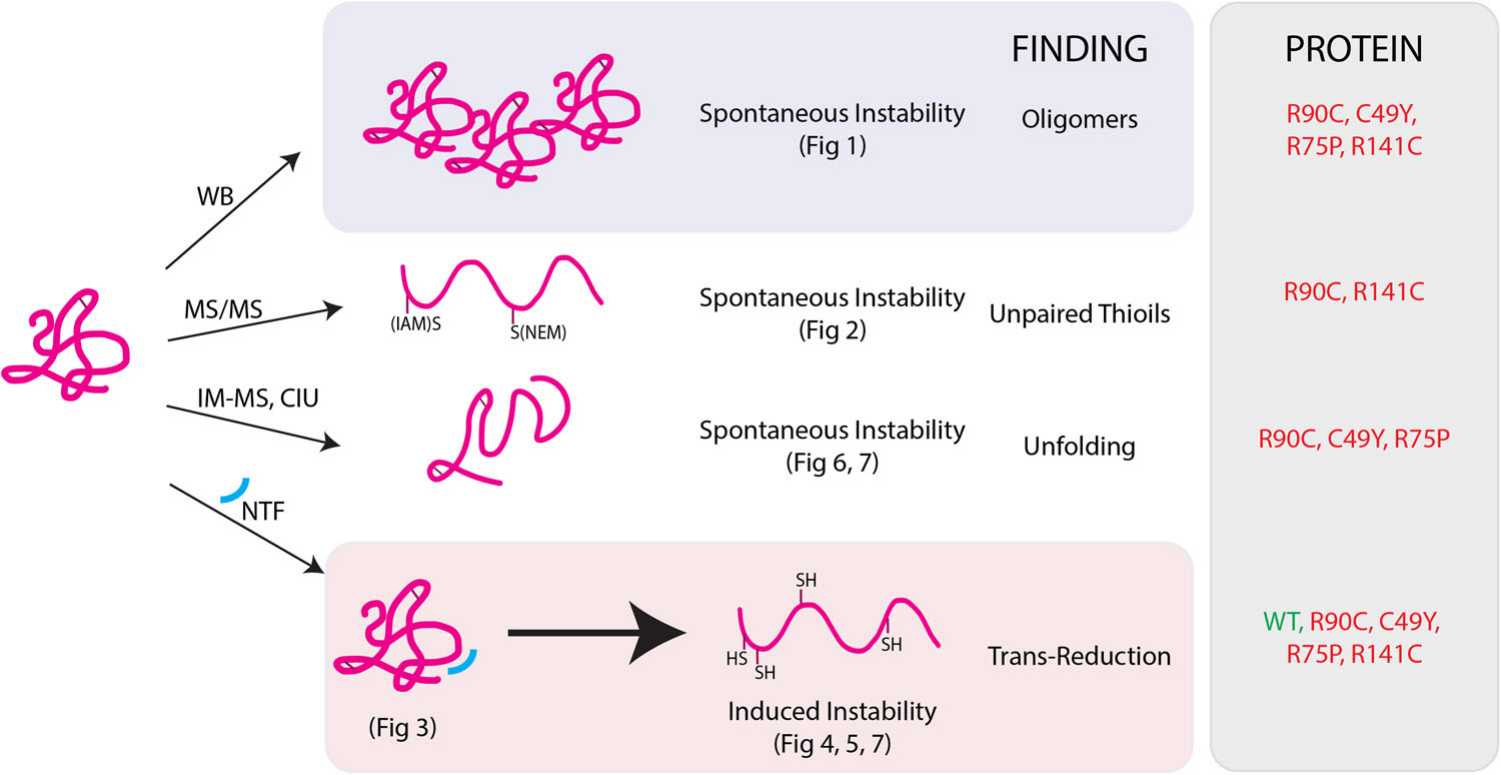
Summary of findings and conclusions. Overall, most mutants demonstrated increased spontaneous instability compared to WT protein, but not all mutants revealed increased instability in all assays tested. Of the assessments of spontaneous instability, multimerization (first row) was observed for all mutants tested. The number of reduced thiols and dynamic instability differed among mutants (second and third rows). The addition of NTF (light blue arc in red box; fourth row), however, induced all mutant proteins into an even less stable state by multiple measures: increased free cysteine labeling visualized by SDS-PAGE gels and MS/MS, increased presence of degradation products identified by IM-MS, and decreased energy requirements for protein unfolding observed by CIU. NTF also altered WT protein but to a lesser degree compared to mutant protein.

Previous work demonstrated that mutant proteins exhibit elevated multimerization. Duering used SIFT to demonstrate that the formation of multimers occurred with mutant NOTCH3^5^. Meng et al., in contrast, showed that both WT and mutant NOTCH3 interact with other NOTCH3 fragments without distinguishing affinity^9^. The current work demonstrates that only a small region of NOTCH3 (the first three EGF-like repeats) is necessary for multimerization, and that the mutations in NOTCH3 that cause CADASIL strongly drive multimerization to a degree unmatched by prior work (Fig. 1c; Fig. 8, blue box). The current study shows that multimerization is dependent on redox status and directly implicates disulfide bonds in the process. The findings are consistent with the possibility that mutant NOTCH3 has a strong propensity for the formation of intermolecular disulfide bonds that bind NOTCH3 proteins together. MS/MS analysis, moreover, shows that an overwhelming majority of cysteines are occupied in disulfide bonds and a minority of cysteines are in the thiol state in only selected CADASIL mutants. This is consistent with the facile formation of inappropriate disulfides at the expense of normal disulfide pairing.

In prior work, we have shown that multimers of NTF are present in CADASIL tissue^12^; moreover, protein multimerization provides an attractive mechanism to explain the dramatic protein accumulation in arteries of CADASIL. This lends weight to CADASIL’s similarity to other degenerative conditions in which multimers of proteins have been the focus of a great deal of attention^2,16^.

The clear differences in disulfide-dependent multimerization demonstrated that at some point in the genesis of mutant proteins, multiple thiols acquired the capacity to react with other cysteines. This was borne out in MS/MS mapping studies which demonstrated increases in thiol cysteine residues in two mutants examined, R90C and R141C (Fig. 2; Fig. 8, second row). This is, to our knowledge, the first direct evidence that CADASIL mutations increase the number of thiol residues, implicating a deficiency in normal disulfide formation in these mutants. However, two additional mutants, R75P and C49Y, did not show elevations in reactive thiols, indicating that disease-causing mutant proteins do not uniformly harbor reactive thiols. While these disease-causing mutations did not alter the number of thiol cysteines, both exhibited extensive multimerization, demonstrating that all tested mutations resulted in abnormal disulfide formation.

The percentage of residues that were in the thiol state in mutants was relatively low (2.00–4.97%), indicating that a high percentage of thiol groups are occupied in disulfide bonds. One explanation for the aggregate findings is that mutant proteins form abnormal disulfide linkages, due to increased thiol availability and that this process continues to progress until all or most of the reduced thiols are occupied in oligomers. The steady-state thiol mapping studies showing specific cysteine residues labeled with NEM indicated that reduced thiols tend to cluster near the site of the mutation, rather than at specific hot spots in the polypeptide. One implication is that these residues represent the areas of mutant protein that are least likely to oxidize via the formation of native disulfide bonds.

The high occupancy rate of thiols of the mutant R90C and R141C cysteine residues deserves attention. These cysteines, which are absent in WT protein, were unavailable to NEM in a very high fraction of proteins (95.24% and 93.17%, respectively). The nearly complete occupancy of these residues suggests that a high proportion of these mutant NOTCH3 proteins contain abnormal disulfide bonds. If this is the case, these bonds could constrain side chains of NOTCH3 that are not normally connected. This is the first indication that we are aware of that specific mutant cysteine residues have been shown to possess abnormal chemical reactivity.

Interestingly, cysteines found to have increased proportions of NEM labeling (and thus inferred to be reduced) were clustered near the location of each respective mutation. However, free cysteines were not limited to the single EGF-repeat containing the mutation, suggesting that disulfide abnormalities induced by CADASIL mutations can span across several EGF-like repeats. This introduces the possibility of a spreading effect, where a single CADASIL mutation can induce additional disulfide changes throughout the protein. Additional studies are required to determine whether CADASIL mutations can induce changes in regions beyond the three EGF-like repeats studied here.

In mapping available thiols in NOTCH3, we uncovered evidence for multiple reduced cysteine residues that were found at a low rate in both WT and mutant proteins. The unequivocal identification of these forms of the protein underscores the molecular heterogeneity of the protein and the potential for a single cysteine abnormality to result in multiple cysteine alterations in a protein. These multiply reduced forms raise the likelihood that NOTCH3 protein may evolve along a continuum of distinct forms with progressively increased aberrant disulfide/thiol content that is influenced by additional factors. The discovery of multiple reduced cysteines in NOTCH3 is consistent with prior work; antibodies that react with diseased CADASIL vessels were shown to react only with NOTCH3 protein that harbored extensive (multiple) cysteines in thiol states^8^. However, it was surprising that the multiple reduced forms were uncommon and not dramatically enriched in mutants.

The relatively low prevalence of unreacted thiols prompted us to consider if pathological NOTCH3 fragments could promote more extensive reduction of protein. Specifically, we considered the possibility that alterations could result from the action of a fragmentation product of NOTCH3, NTF, found in diseased CADASIL vessels^11^. In this study, we provide evidence suggesting physical interaction between NTF and NOTCH3 in vitro and in vivo (Figs. 3, 7a). We further demonstrate that NTF can reduce disulfides in trans in NOTCH3, (trans-reduction; (Fig. 4; Fig. 8, red box)), providing a potential mechanism through which multiple reduced cysteines in NOTCH3 are formed in CADASIL.

We observed higher sensitivity to trans-reduction in mutants compared to WT (Fig. 4). However, we did not observe this difference with MS/MS (Fig. S5). The discrepancy between the two detection methods could be explained in part by the additional protein processing required for MS/MS experiments. Notwithstanding, the MS/MS finding that NTF affects WT and mutant NOTCH3 protein across similar residues, regardless of the type and location of mutation could explain why hundreds of different mutations can lead to similar clinical presentations (Fig. S5). Perhaps, the presence of disease-specific molecules, such as NTF, is the key factor required to reduce NOTCH3 and initiate disease processes. Despite these differences in WT vs. mutant protein susceptibility to NTF, we can conclude from our studies that NTF is capable of reducing NOTCH3 at the cysteine level and that under the conditions we used, NTF does not preferentially affect specific regions of NOTCH3. That NTF also affects WT protein suggests that it could be implicated in mechanisms of the more common sporadic small vessel disease, in addition to mechanisms of inherited small vessel disease.

Three of four mutants demonstrated decreased protein stability by IM-MS and by CIU. The identification of increased fragmentation in select mutants was similarly visualized on nonreducing western blot experiments after 1-h incubation at 37 °C (Fig. S3a). In prior work, CIU fingerprint features have been correlated to the number and patterns of disulfide bonding to study their effects on protein stability^15^. Our CIU studies support that the destabilization of proteins in the presence of disease-causing mutations and disease-specific molecules (NTF) likely occurs through biochemical alterations in disulfide bonding. Our studies support a mechanism of both inherent and *trans*-induced NOTCH3 instability in disease, which may contribute to the hallmark protein alterations observed in the disease.

The findings of this study require additional follow-up. For example, to facilitate protein production and analysis, the investigations were confined to the first three EGF-like domains of NOTCH3. Yet, CADASIL mutations are distributed widely throughout the entire ectodomain, and, as such, it will be important to understand if the alterations in structure and stability discovered here in the N-terminus are reflected elsewhere or in larger fragments of recombinant protein. The emerging recognition that mutation location may affect severity of phenotypes^17^ indicates that future study of other regions of NOTCH3 may reveal a correlation between the degree of biochemical changes and clinical features. This study also suggests that a non-cysteine CADASIL mutations in NOTCH3 (R75P) is sufficient to induce similar alterations in multimerization, thiol lability, and instability as canonical cysteine CADASIL mutations. Extension of the experiments to additional non-cysteine mutations may help elucidate the structural requirements underlying CADASIL proteinopathy^18,19^.

Overall, these results highlight, to the best of our knowledge, a multitude of novel biochemical differences between WT and mutant NOTCH3 (Fig. 8). Some of these changes occur only in a subset of mutants, but three differences are shared by all mutants: oligomerization, susceptibility to trans-reduction by NTF, and destabilization by trans-reduction. Due to their convergence between all mutants, these features are strong candidates for molecular drivers of CADASIL pathogenesis.

## Methods

### Chemicals and reagents

Unless otherwise noted, chemicals were purchased from Sigma and cell culture reagents were purchased from Invitrogen.

### DNA Constructs and recombinant NOTCH3 protein generation

Fragments of NOTCH3 cDNA were fused to mouse Fc (IgG) in frame to produce recombinant NOTCH3 proteins. NOTCH3 fragments, derived from full-length NOTCH3 were generated using PCR with restriction recognition sequence engineering into primers to enable standard ligation-mediated cloning, as previously described^9^. All plasmids were sequenced to confirm coding sequences. Cell lines stably generating recombinant NOTCH3 proteins were generated, and proteins were purified as previously described^9^. Primers used for PCR were synthesized by IDT; sequences used to amplify the 5′ and 3′ cloning junctions included restriction sites and were: sense primer for mouse Fc (5′-GGCGCGCCCCCAGAGTGCCCATAACACAGAACCCC-3′), anti-sense primer for mouse Fc (5′-AAGCTTTTTACCCAGAGACCGGGAGATGGT-3′), sense primer for NOTCH3 open reading frame (5′-GGCGCGCCAAGCTTGCCCCCCCTTGCCTGGACGGAAGC-′3) anti-sense primer for NOTCH3 open reading frame (5′-CTCGAGTCACTCATCCACGTCGCTTCGGCAGCT-3′).

### Cell Culture, transfections, and immunoprecipitation

HEK 293 (QBiogene) cells were grown in Dulbecco’s modified Eagle’s medium (Invitrogen) with 10% fetal bovine serum. Human HEK293 cells were grown to over 70% confluence and then transfected using Lipofectamine 2000 (ThermoFisher) or PolyJet (SignaGen) according to the manufacturer’s instructions. Protein A agarose was used to pull down immune complexes, which were then analyzed by immunoblotting.

### Protein analysis and western blotting

Proteins were either denatured in sample buffer containing beta-mercaptoethanol or nonreducing sample buffer and boiled at 100 °C for 3 min. All samples were separated on standard 10% or gradient 4–20% SDS-PAGE gels (ThermoFisher) and electroblotted to nitrocellulose using an iBlot 2 system. Western blot analysis was performed with antibodies as indicated, followed by incubation with infrared fluorophore-labeled secondary antibodies (Rockland). Bands were detected using a Li-Cor Odyssey infrared scanner.

For cysteine-labeling western blot experiments, thiols were labeled with 10 mM NEM (Sigma) for 3 h RT in the dark or 10uM IRDYE 800CW Maleimide (Li-Cor) for 30 min RT in the dark. Proteins were separated using 4–20% SDS-PAGE gels (ThermoFisher) and IRDYE 800 signal was detected using a Li-Cor infrared Odyssey scanner. NEM labeling was detected using an antibody specific for NEM labeled proteins, OX133 (Absolute Antibody). Relative protein amount was normalized to signals obtained using secondary antibodies against mouse Fc.

NTF challenges were performed by mixing synthetic NTF peptides with recombinant proteins as described in individual experiments; NTF was composed of a 41 amino acid sequence validated by mass spectrometry as described before^12^.

### Protein identification by LC-tandem mass spectrometry

WT and CADASIL mutant protein samples were purified, as described earlier^9^, sequentially labeled with two cysteine-labeling compounds (N-ethylmaleimide and 2-chloroacetamide) before and after chemical reduction with DTT, and submitted for LC-Tandem-MS at the Mass spectrometry Facility of the Department of Pathology at the University of Michigan. See supplemental information (SI) for experimental details. The mass spectrometry proteomics data have been deposited to the ProteomeXchange Consortium via the PRIDE partner repository with the dataset identifier PXD031097^20^.

### Collision-induced unfolding

*Sample preparation*. Purified Fc-Notch3 constructs (~5 μM) were dialyzed into 200 mM ammonium acetate solution. Samples were concentrated using 10 kDa MWCO Amicon Ultra-0.5 Centrifugal Filter Unit (Millipore Sigma) at 4 °C.

*Native ion mobility-mass spectrometry*. Samples were analyzed using a quadrupole ion mobility time-of-flight mass spectrometer (Q-IM-TOF SELECT SERIES Cyclic IMS; Waters Corporation, Milford, MA)^21,22^. See SI for additional information on instrumentation.

Mass spectra and drift time distributions were obtained for ions at collision energies from 5 to 190 V in 5 V increments. Each drift time distribution for the +20-charge state of Fc-Notch3 constructs was extracted by using TWIMExtract v1.6^23^ and analyzed in CIUSuite2 v2.2^24^. See SI for analysis methods.

### Proximity ligation assay

Formalin-fixed frontal lobe sections were obtained from the Alzheimer’s Disease Center at the University of Michigan and the Brain Bank of the National Institute for Developmental and Childhood Disorders at the University of Maryland. CADASIL frontal lobe tissue obtained at autopsy has been previously described^25–27^. Five-micron sections were prepared for PLA using standard immunohistochemical methods. PLA was performed according to the manufacturer’s brightfield PLA instructions, although we increased the amplification time to 5 h (Sigma Aldrich).

### Statistics and reproducibility

Significant differences were determined using unpaired two-tailed Student’s *t*-test on GraphPad Prism v.7.0 c. A *p* value < 0.05 was considered to be statistically significant.

Statistical analyses of two populations with binomial distributions were done by calculating the test-statistic, z-score, using the following equations:

1 $$z=\frac{\bar{p1}-\bar{p2}}{\sqrt{\bar{p}\left(1-\bar{p}\right)}\left(\frac{1}{n1}+\frac{1}{{n}_{2}}\right)}$$

where

2 $$\bar{p}=\frac{{n}_{1}{p}_{1}+{n}_{2}{p}_{2}}{{n}_{1}+{n}_{2}},$$

*n*_1_ refers to the total number of measurements for sample 1, *n*_2_ refers to the total number of measurements for sample 2, *p*_1_ refers to the average probability for proteins in sample 1 to be labeled with NEM and *p*_2_ refers to the average probability for proteins in sample 2 to be labeled with NEM. The differences in probabilities were plotted (Figs. 2, 5) and the calculated Z-scores were correlated to critical region values to obtain *p*-values using an online normal distribution calculator (http://www.distributome.org/V3/calc/NormalCalculator.html).

### Reporting summary

Further information on research design is available in the Nature Research Reporting Summary linked to this article.

## Supplementary information

Supplementary Information

Reporting Summary

## Data Availability

All data generated or analyzed during this study are included in this published article and its supplementary information files. All unprocessed western blots can be found in Supplemental Information, Figs. S8–S12. All plasmids will be deposited into Addgene under deposit number 80617. All proteomics MS/MS data have been deposited into PRIDE^28^, project accession ID: PXD031097. All source data have been deposited into figshare (10.6084/m9.figshare.18214655)^29^.
