# Supplementary material for: Oligomerization, trans-reduction, and instability of mutant NOTCH3 in inherited vascular dementia
Source: Commun Biol. 2022 Apr 7;5:331. doi: 10.1038/s42003-022-03259-2 (PMC8991201; doi:10.1038/s42003-022-03259-2)
Supplement: Supplementary file 1 — Supplementary Information [file 42003_2022_3259_MOESM1_ESM.pdf]

SUPPLEMENTAL INFORMATION

Supplemental Methods

Protein Identification by LC-Tandem MS.

In solution digestion. 5ug of WT and CADASIL mutant protein samples were purified, as described earlier[9]. Cysteines were labeled with 10mM NEM and unreacted NEM was removed by dialysis. Protein samples were submitted for further processing and analysis at the Mass spectrometry Facility of the Department of Pathology at the University of Michigan.

Cysteines were reduced by adding 10mM DTT and incubating at 45ºC for 30 minutes. Samples were cooled to RT and alkylation of cysteines was achieved by incubating samples with 65mM 2-chloroacetamide, under darkness, for 30 minutes at RT. An overnight digestion with sequencing grade, modified trypsin (enzyme: substrate ratio of 1:50; Promega) was carried out at 37ºC with constant shaking in a Thermomixer. Digestion was stopped by acidification and peptides were desalted using SepPak C18 cartridges according to manufacturer's protocol (Waters). Samples were completely dried using vacufuge (Eppendorf).

In-gel digestion. NTF was added to 5ug of WT and CADASIL mutant protein samples at a 25:1 molar ratio for 1 hour at 37ºC. Cysteines were labeled with 10mM NEM and samples were separated by SDS-PAGE and stained with SimplyBlue SafeStain (ThermoFisher). NOTCH3 bands were excised and submitted for processing and analysis at the Mass Spectrometry Facility of the Department of Pathology at the University of Michigan. Gel slices were destained with 30% methanol for 4 hours. Upon reduction (10mM DTT) and alkylation (65mM 2-Chloroacetamide) of the cysteines, proteins were digested overnight with 500ng of sequencing grade, modified trypsin (Promega) at 37ºC. Peptides were extracted by incubating the gel with 150uL of 50% acetonitrile/0.1% TFA for 30 minutes at RT. A second extraction with 150uL of 100% acetonitrile/0.1% TFA was also performed. Both extracts were combined and dried in a vacufuge (Eppendorf).

MS/MS. Resulting peptides were dissolved in 9uL (for in-gel digest) or appropriate volume of 0.1% formic acid/2% acetonitrile solution (to achieve ~500ng peptide/uL) and 2uLs of the peptide solution were resolved on a nano-capillary reverse phase column (Acclaim PepMap C18, 2 micron, 50cm, ThermoScientific) using a 0.1% formic acid/ 2% acetonitrile (Buffer A) and 0.1% formic acid/ 95% acetonitrile (Buffer B) gradient at 300nL/min over a period of 90 min (2-25% Buffer B in 45 min, 25-40% in 5 min, 40-90% in 5 min followed by holding at 90% Buffer B for 5 min and re-equilibration with Buffer A for 30 min). Eluent was directly introduced into *Q exactive HF* mass spectrometer (Thermo Scientific, San Jose CA) using an EasySpray source. MS1 scans were acquired at 60K resolution (AGC target=$3x10^6$; max IT=50ms). Data-dependent collision induced dissociation MS/MS spectra were acquired using Top speed method (3 seconds) following each MS1 scan (NCE~28%; 15K resolution; AGC target $1x10^5$; max IT 45ms).

Database Search. Proteins were identified by searching the data against a custom protein database containing WT and mutant NOTCH3 protein and NTF fragment appended with common contaminant protein list (ftp://ftp.thegpm.org/fasta/cRAP) using Proteome Discoverer (v2.4, Thermo Scientific). Search parameters included MS1 mass tolerance of 10ppm and fragment tolerance of 0.1 Da; two missed cleavages were

allowed; carbamidomethylation and N-ethylmaleimide modification of cysteine, oxidation of methionine, and deamidation of asparagine and glutamine, were considered as potential modifications. False discovery rate (FDR) was determined using Percolator and proteins/peptides with FDR of ≤1% were retained for further analysis.

Collision Induced Unfolding.

Native Ion Mobility-Mass Spectrometry. Sample (8uL) was transferred to a gold-coated borosilicate capillary (0.78 mm i.d., Harvard Apparatus, Holliston, MA) for direct infusion by nano-electrospray Ionization (nESI). Protein ions were gently transferred into the gas-phase without activation by using the following optimized instrument parameters: a capillary voltage of 1.2 -1.4 kV was used along an extraction cone voltage of 70V. The source temperature was kept at 30˚C for proper desolvation without denaturation. Ion Guide Gas was 35ml/min, while helium and nitrogen gas flows were 150 and 45 mL/min, respectively. Backing, Source, Trap and TOF pressures were 8.67 x10^{-3}, 2.44x10^{-2} mbar and 4.42 x10^{-7} mbar, respectively. The cyclic IM racetrack travelling settings were a wave velocity 375 m/s with wave height of 38 V under a race bias of 70 V. Cyclic separation sequence includes injection (5ms), separate (5 ms), and eject and acquire. PreArray Gradient is 95 (injection) and 85 (separate, eject and acquire). Pre-Array bias is 80 (injection) and 70 (separate, eject and acquire). Racetrack Entrance is 10 (inject), 30 (separate) and 50 (eject and acquire). Racetrack exit is 50 (inject), 30 (separate) and 2 (eject and acquire). Offset is exit is 60 (inject), 70 (separate) and 60 (eject and acquire). Wave map is 8 (inject), 0 (separate) and 25 (eject and acquire). Post Array Gradient and Bias were 35 and 10 for the whole cyclic sequence.

Data Analysis. Each drift time distribution intensity was normalized to one and plotted as a function of collision voltage to generate a CIU fingerprint. Fingerprints were smoothed using Savitzky-Golay method with a window size of 5 and 3 iterations. Features and CIU-50 calculations were used with the respective CIUSuite2 modules. For feature detection a maximum feature length of 4 was used, along a Feature Allowed width of 1.15 units and Maximum Gap Length of 5V steps. IM-MS data was visualized by MassLynx v4.2 and Driftscope v3.0 from Waters Corporation.

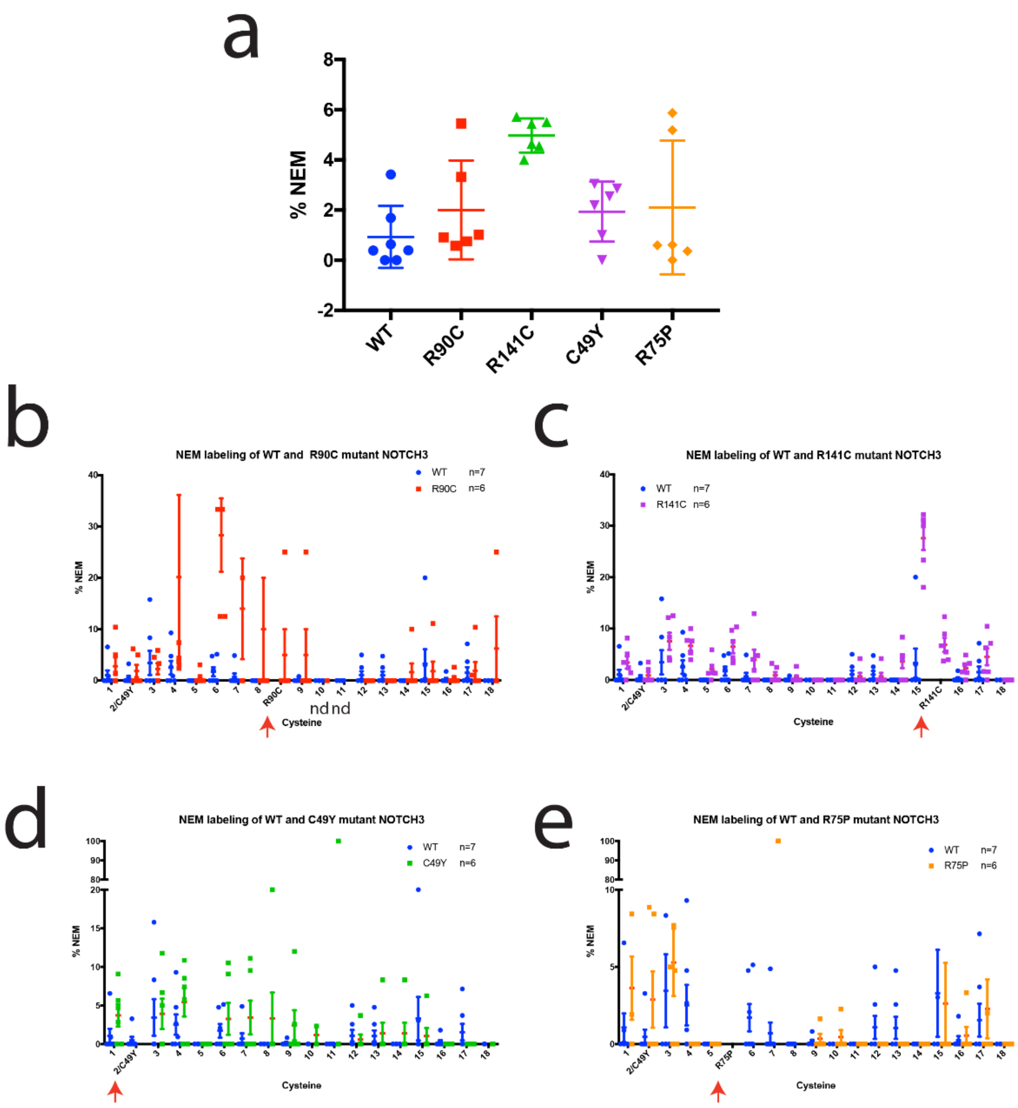

Figure S1: *Select mutant NOTCH3 proteins have increased free thiols available for NEM labeling compared to WT NOTCH3.* Purified WT and mutant Fc-NOTCH3 protein were labeled with NEM, reduced with DTT, labeled with 2-Chloroacetamide, trypsinized, and examined with MS/MS. Global NEM content was measured from at least 6 replicate experiments and displayed in [a]. We observed increased NEM content in mutants compared to WT [a, ns]. Proportion of NEM labeled cysteines out of total cysteines was calculated from peptide spectrum matches (PSMs) and displayed in [b-e]. [b] shows increased amounts of NEM label in R90C mutant protein compared to WT protein, with greatest amounts of NEM labeling in cysteines 6-9. [c] shows increased amounts of NEM label in R141C mutant protein compared to WT protein, with the greatest amount of NEM labeling found at cysteine position 15. [d] compares NEM labeling between C49Y mutant protein and WT protein and [e] compares NEM labeling between R75P mutant protein and WT protein. Slightly increased NEM labeling was observed in C49Y and R75P mutant proteins compared to WT. Each experiment was repeated at least 6 times with similar results. Figure S1 demonstrates data from all available replicates. Locations of mutations are marked with a red arrow and cysteines that were not detected were noted with “nd.”

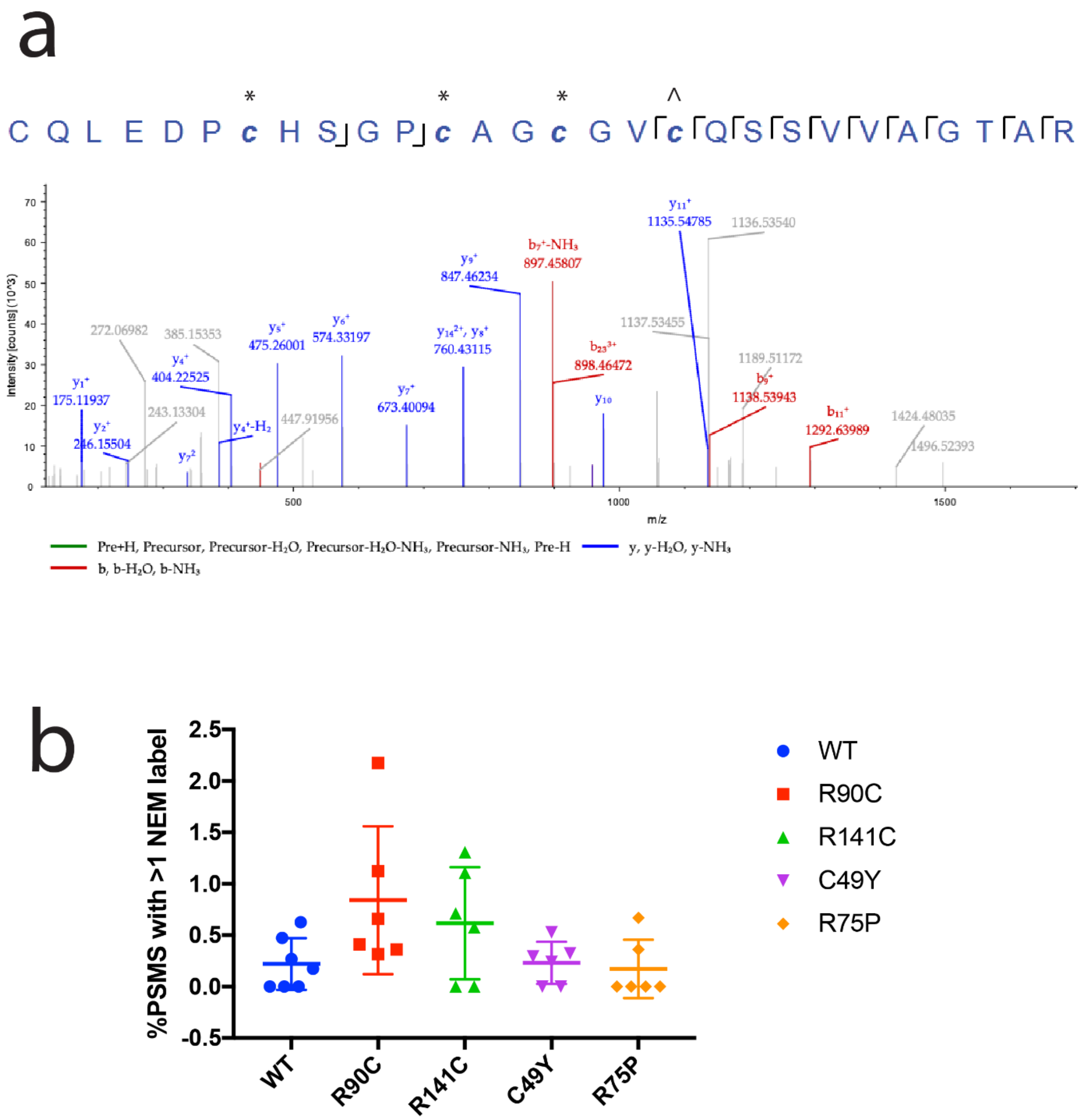

Figure S2: *Single tryptic fragments occasionally have more than one cysteine available for NEM labeling.* Tryptic parent fragment masses were measured and numbers of cysteine labels [NEM (*) or 2-Chloroacetamide (^)] were determined. Further fragmentation of each peptide into b- and y-ions allowed for assignment of each label to a specific cysteine, when possible. Several parent fragments included multiple cysteines labeled with NEM (*). [a] demonstrates an example spectra of a single parent fragment from R90C mutant protein with 3 NEM labels. Examination of both WT and mutant proteins revealed fragments with multiple NEM labeled cysteines. The proportion of peptide spectrum matches (PSMs) with >1 NEM label within the total number of PSMs was slightly increased in the R90C and R141C mutant proteins [ns, b]. Experiments were done at least 6 times with similar results.

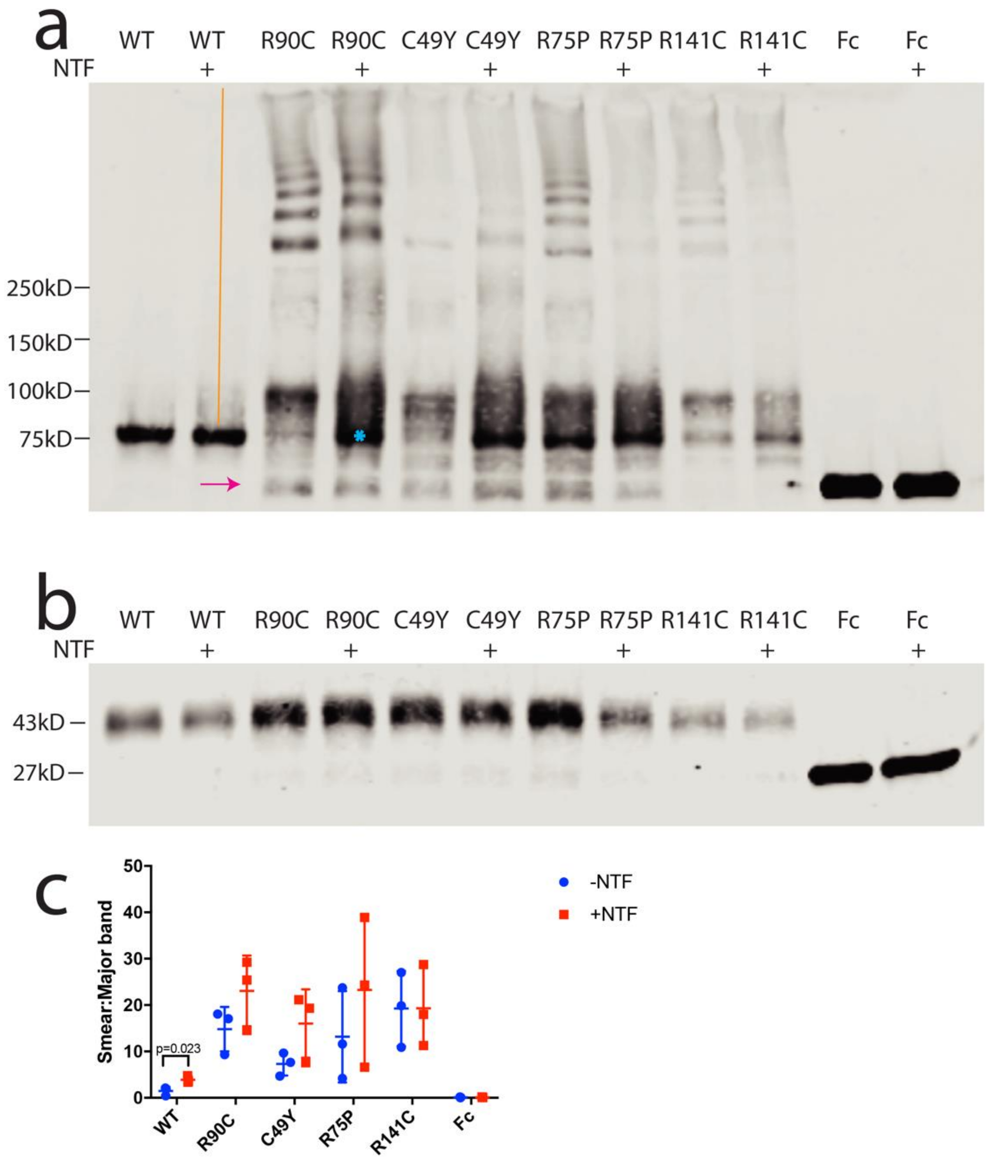

Figure S3: *NTF addition alters the mobility pattern of mutant NOTCH3 multimers on nonreducing SDS-PAGE gels.* NTF was added to purified Fc-tagged NOTCH3 containing the first 3 EGF-like repeats at a 50:1 ratio for 1hr. Protein was separated on a nonreducing 4-20% SDS-PAGE gel and transferred to nitrocellulose using an iBlot 2 system. Purified NOTCH3 was identified by probing for the Fc tag using a Li-Cor Odyssey instrument. NTF addition resulted in increased smearing in all proteins (vertical orange line) and an upward shift in laddering observed in all mutant proteins [a]. NTF additional also altered the mobility pattern of mutant proteins [a, blue asterisk]. All mutants also demonstrated the presence of a degradation product [a, pink arrow]. Total protein was quantified on a parallel reducing SDS-PAGE gel [b]. Quantification of smearing observed on nonreducing gel [a, orange line] normalized to total protein content [b] revealed significantly increased smearing of WT protein with NTF and a trend towards increased smearing of all mutant proteins [c]. Addition of NTF to Fc tag alone did not alter the mobility pattern [a] or smearing [c]. Unprocessed western blots can be found in Fig S11.

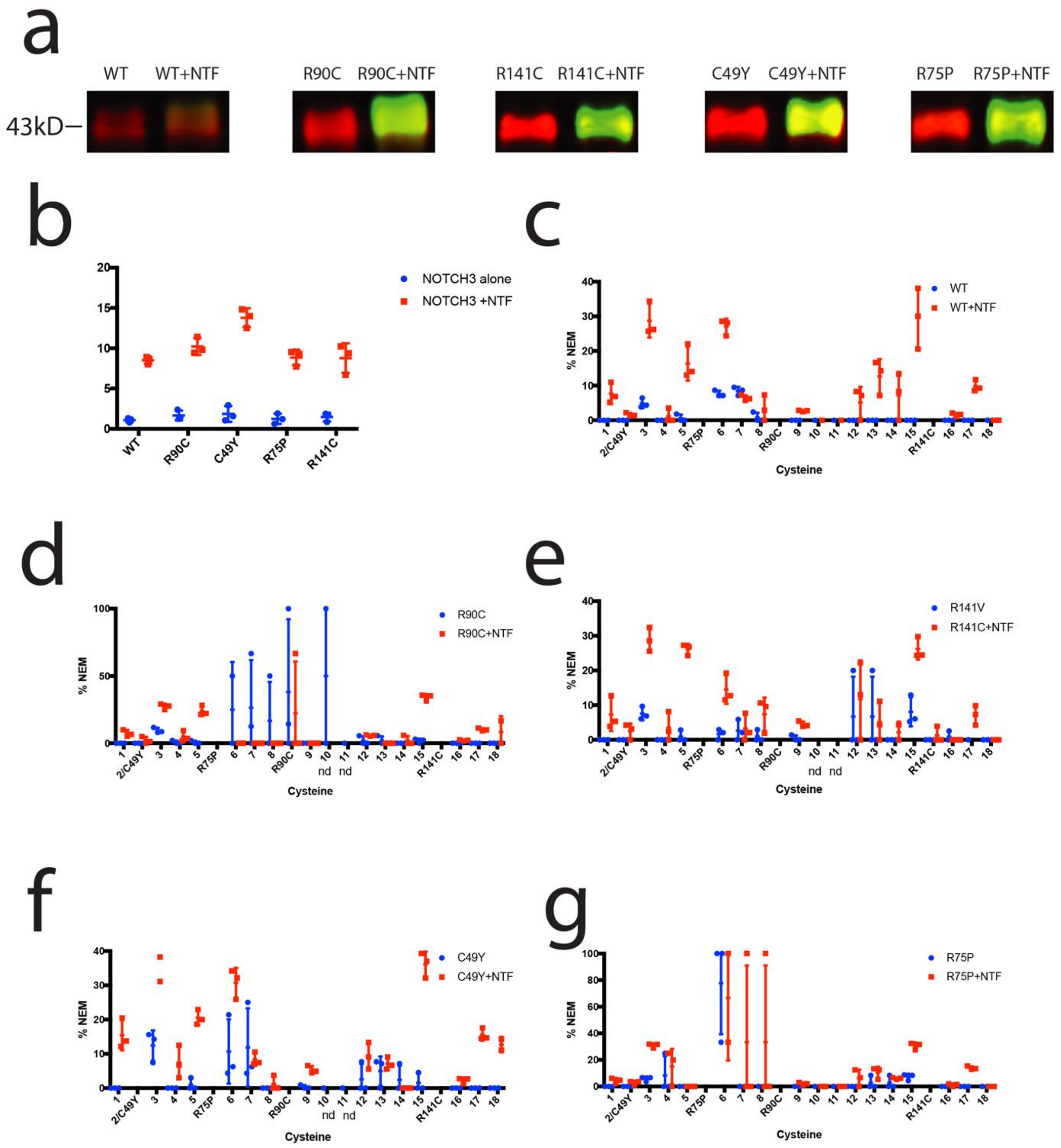

Figure S4: *NTF addition to NOTCH3 liberates cysteines in NOTCH3 detectable by MS/MS.* NTF was added to purified WT and mutant Fc-NOTCH3 protein at a 25:1 molar ratio for 1 hour at 37ºC, labeled with NEM and separated on a 4-20% SDS-PAGE gel (ThermoFisher). A fraction of the reacted protein was first separated on 4-20% SDS-PAGE gels (ThermoFisher) and probed with an antibody against NEM-labeled proteins (OX133, Absolute Antibody[12]). [a] shows increased reactivity of OX133 with NTF treated WT and mutant proteins. The unprocessed western blot can be found in Fig S12. The remainder of the samples were separated on 4-20% SDS-PAGE gels and total protein content was visualized with SimplyBlue Safestain (ThermoFisher). Bands of sizes corresponding to NOTCH3 alone were excised, and the proteins were reduced with DTT,

labeled with 2-Chloroacetamide, trypsinized, and examined with MS/MS. Global NEM content was measured from at least 3 replicate experiments and displayed in [b]. We observed significantly increased NEM content in all samples containing NTF compared to those without NTF ($p<0.001$). We then calculated the proportion of NEM labeled cysteines out of total cysteines from peptide spectrum matches (PSMs) between NOTCH3 with and without NTF and displayed the comparisons for each WT or mutant protein in [c-g]. No PSMs specific for NTF alone were detected. [c] demonstrates increased amounts of NEM label in WT protein with addition of NTF compared to WT protein alone throughout the whole protein fragment. [d] shows increased amounts of NEM label in R90C mutant protein with addition of NTF compared to without NTF at specific regions but decreased labeling at cysteine positions 6, 7, and 8. [e] compares NEM labeling between R141 mutant protein with NTF and R141C mutant protein alone. We found increased NEM labeling in the R141C protein with NTF in most regions but decreased NEM labeling at cysteine position 16. [f] compares NEM labeling between C49Y with and without NTF addition and increased proportions of NEM labeled cysteines were observed in the presence of NTF throughout the protein, with only decreased NEM labeling observed at cysteine position 7. Finally, [g] compares NEM labeling between R75P mutant protein with and without NTF. Increased NEM labeling was observed throughout the R75P mutant protein with NTF condition compared to R75P mutant protein alone, with only slight decrease in NEM labeling observed at cysteine position 6. Each experiment was repeated three times with similar results. Figure S4 demonstrates data from all available replicates. Locations of mutations are marked with a red arrow and cysteines that were not detected were noted with “nd.”

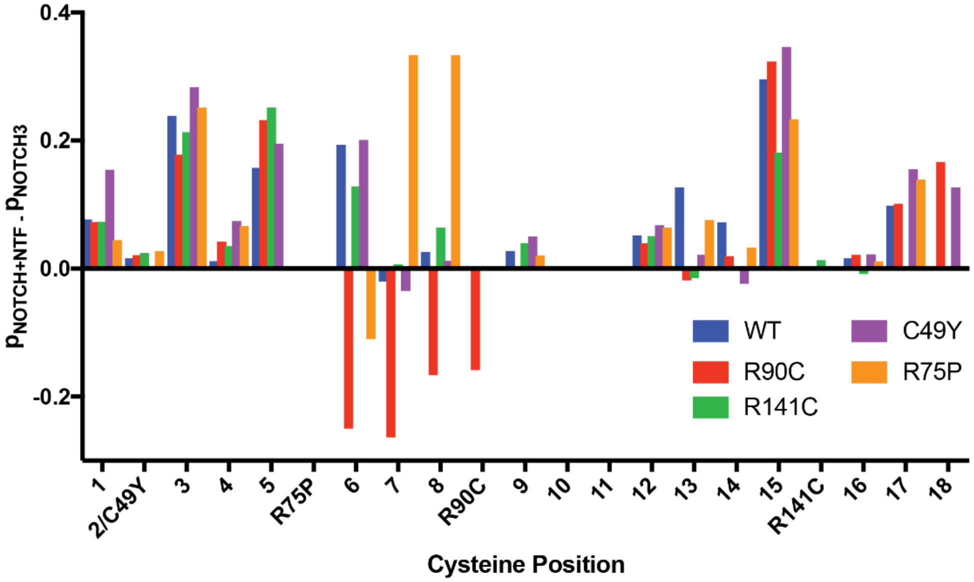

Figure S5: *Most regions of NEM labeling were similar between NTF-treated WT and mutant NOTCH3.* Comparison of regional specificity of NTF-induced NEM labeling changes highlights that WT and mutant protein behaved similarly at most cysteine positions. However, several mutants had regions of significantly decreased NEM labeling with addition of NTF while the WT protein did not.

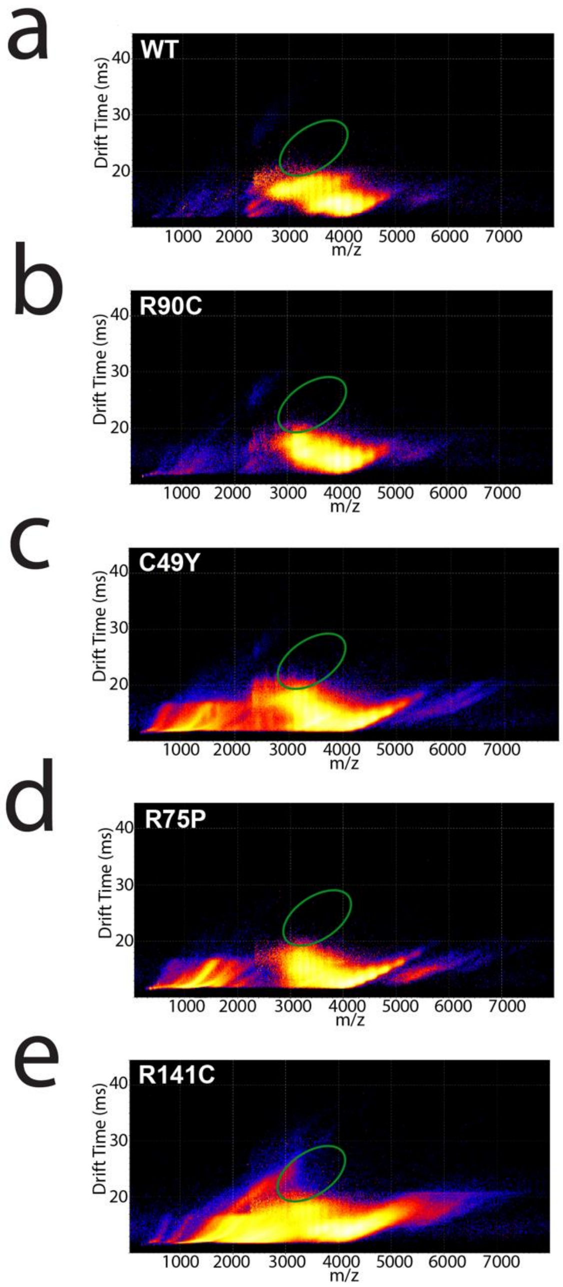

Figure S6: *IM-MS plots of Fc-NOTCH3 constructs at 40V failed to identify a population of degradation product (green circle; Fig 6).* [a] shows an IM-MS plot of WT Fc-NOTCH3, [b] R90C Fc-NOTCH3, [c] C49Y Fc-NOTCH3, [d] R75P Fc-NOTCH3, and [e] R141C Fc-NOTCH3.

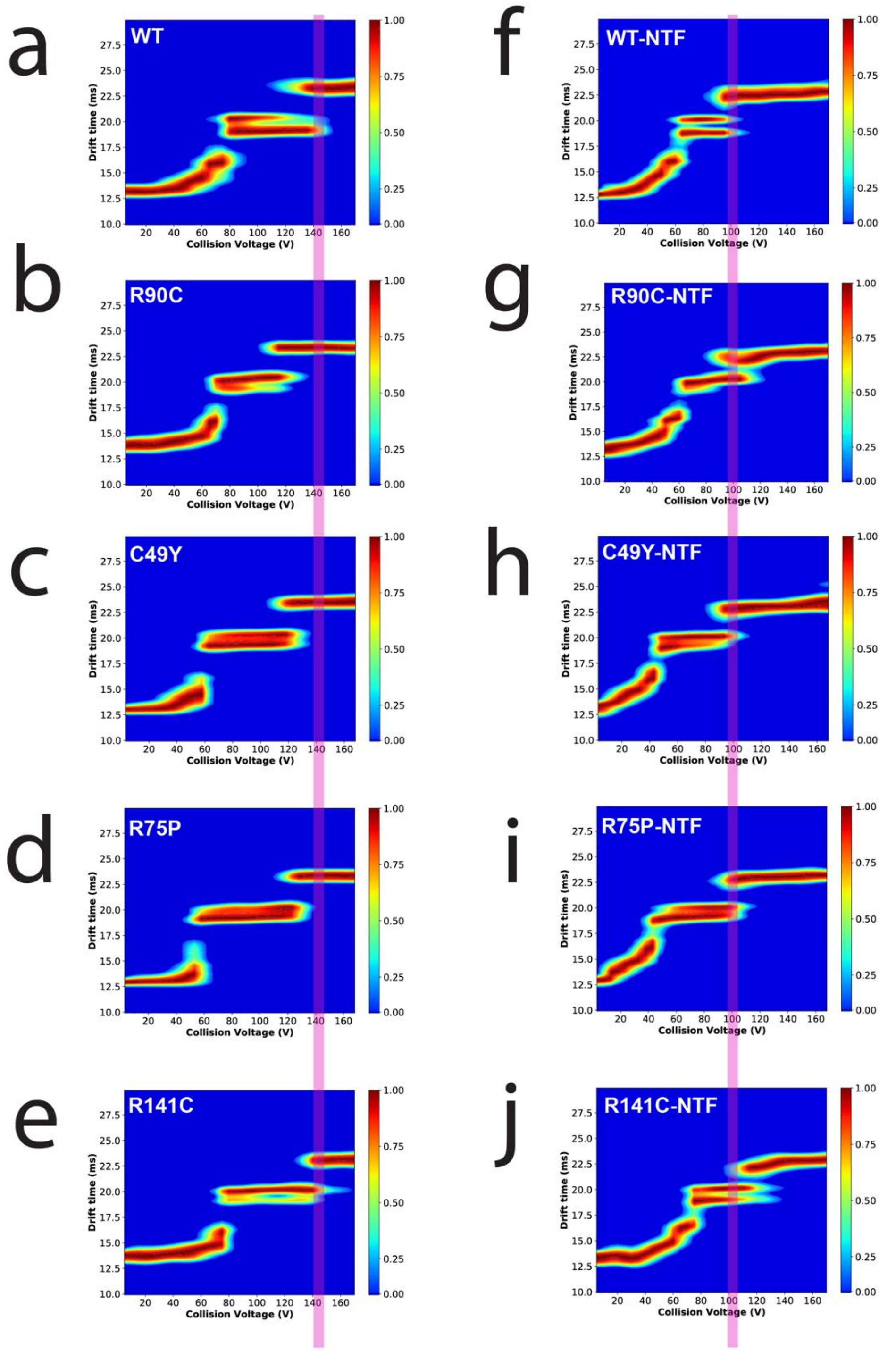

Figure S7: *CIU fingerprints of Fc-NOTCH3 constructs.* The purple bar indicates the onset voltage of the 22.5ms feature relative to WT. We compared the onset voltage between mutants and WT proteins, without NTF addition [a–e] and with NTF addition [f–j].

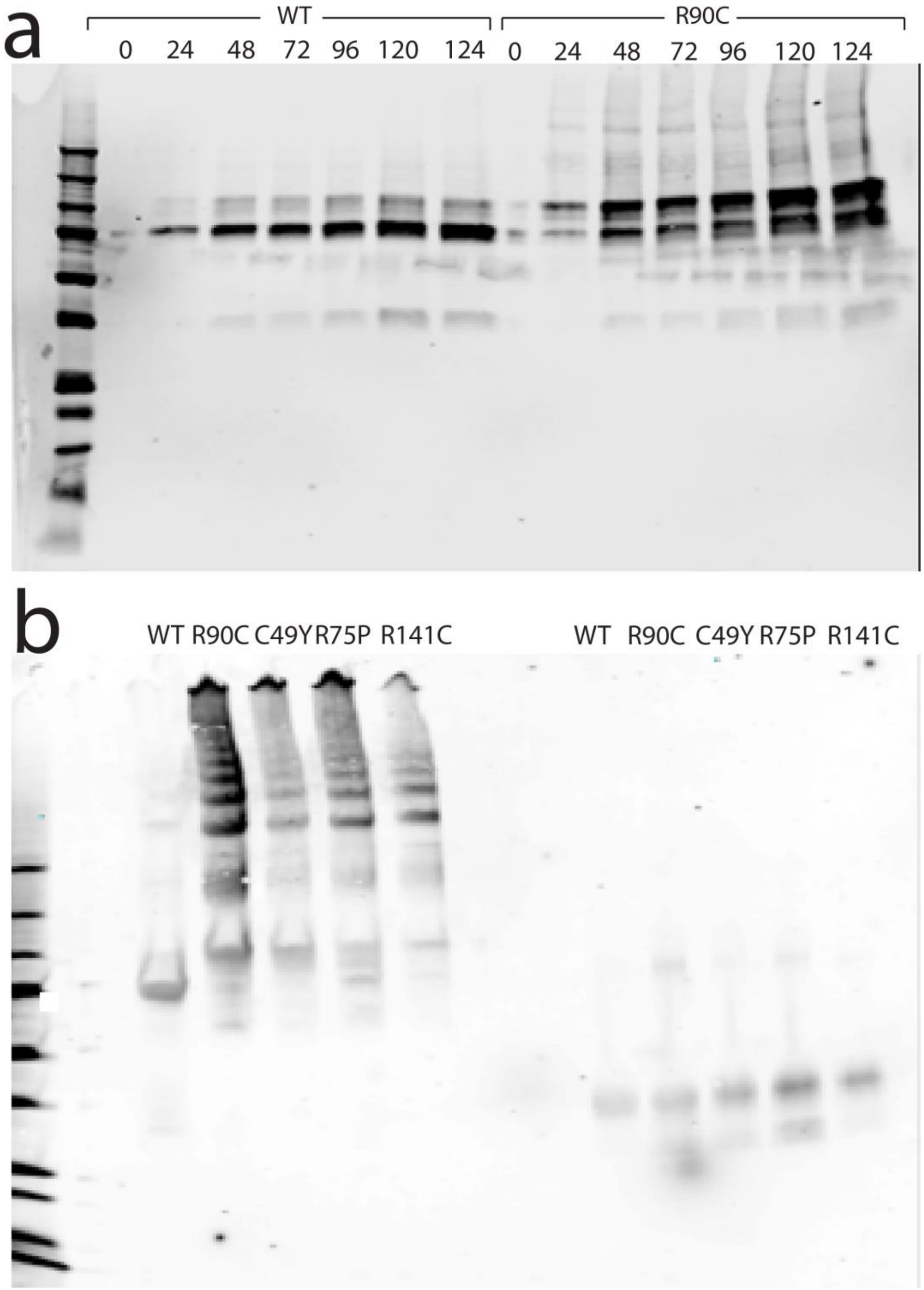

Figure S8: *Unprocessed western blots for Fig 1.* [a] corresponds to Fig 1a and [b] corresponds to Fig 1c-d.

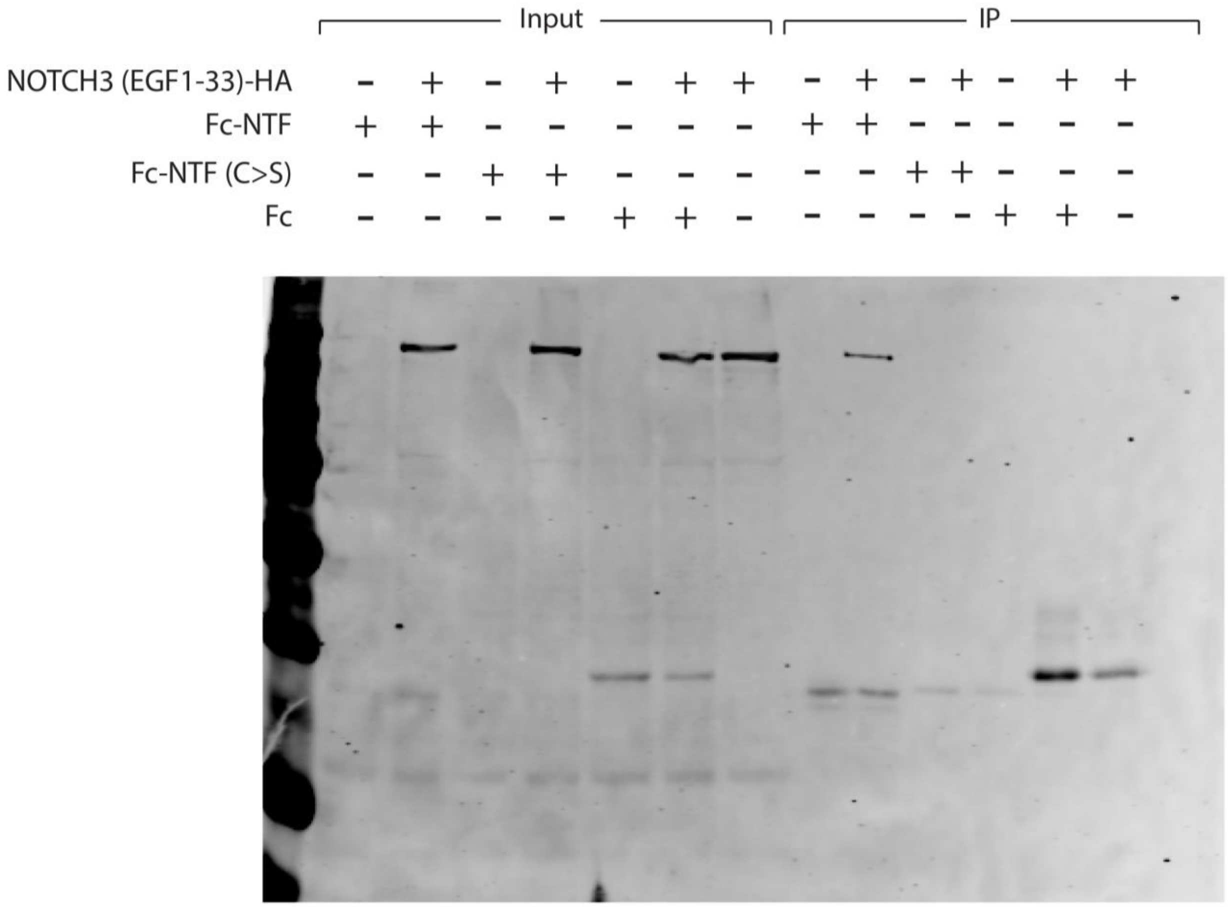

Figure S9: *Unprocessed western blot for Fig 3.*

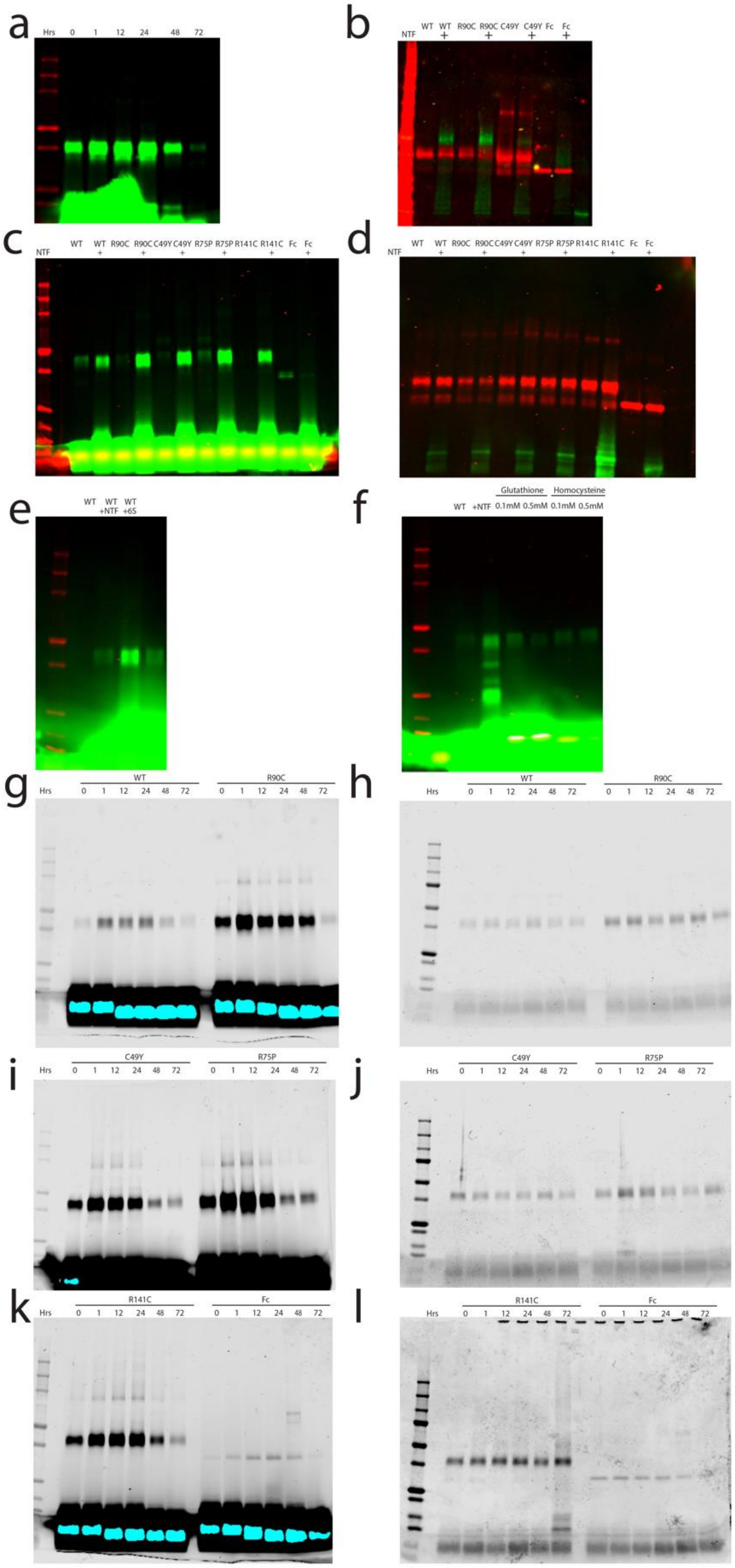

Figure S10: *Unprocessed western blots for Fig 4.* [a] corresponds to Fig 4a, [b] corresponds to Fig 4b, [c-d] corresponds to Fig 4c, [e] corresponds to Fig 4E, [f] corresponds to Fig 4f, and [g-l] corresponds to Fig 4g.

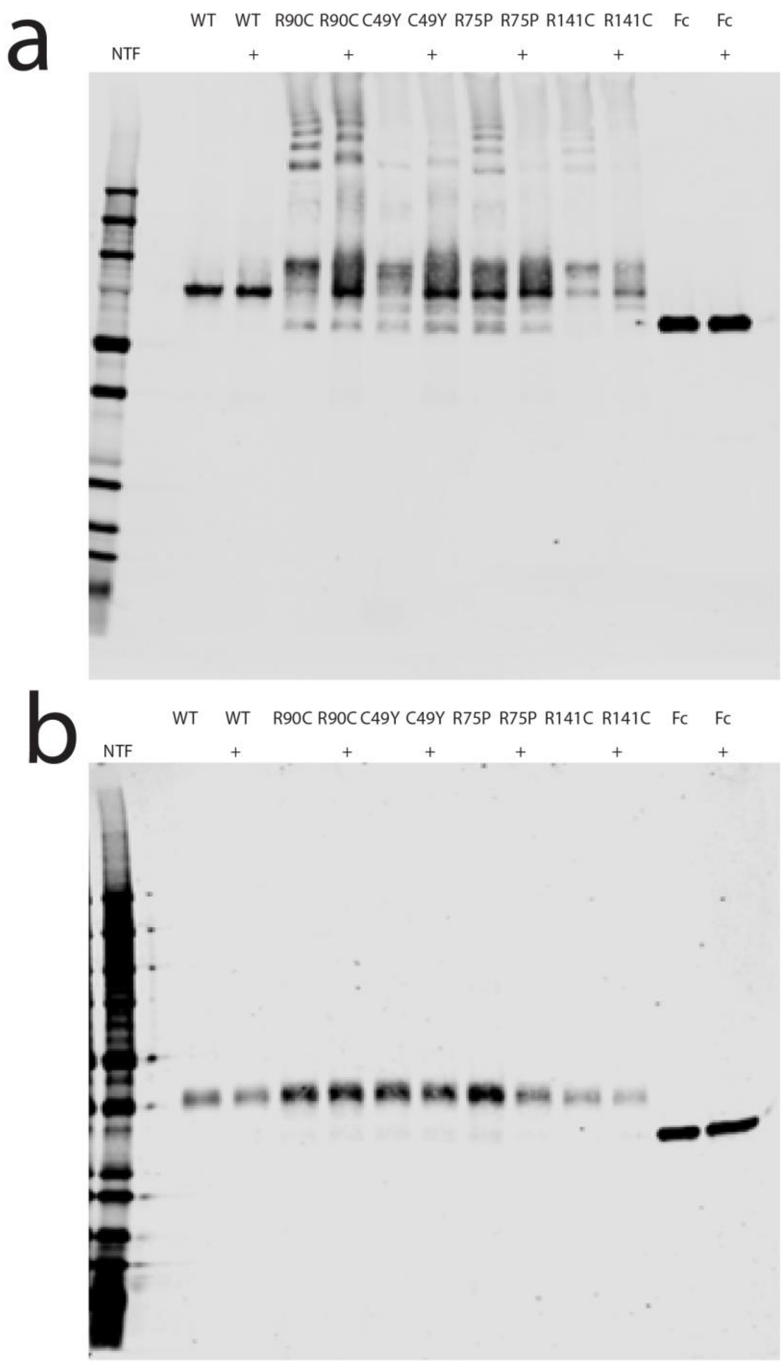

Figure S11: *Unprocessed western blots for Fig S6.* [a] corresponds to Fig S6a and [b] corresponds to Fig S6b.

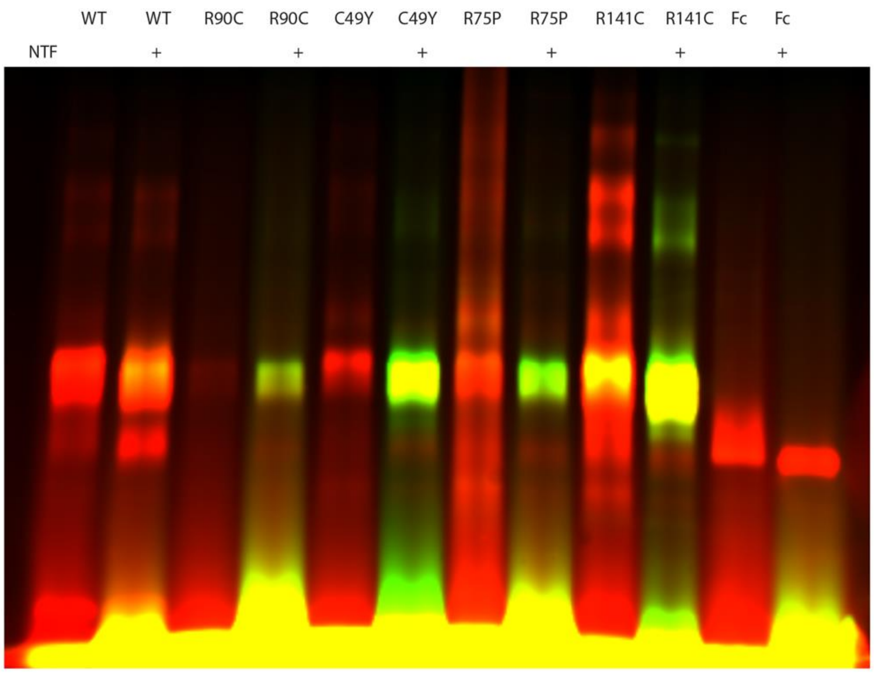

Figure S12: *Unprocessed western blot for Fig S8a.*
