# Supplementary material for: Oligomerization, trans-reduction, and instability of mutant NOTCH3 in inherited vascular dementia
Source: Commun Biol. 2022 Apr 7;5:331. doi: 10.1038/s42003-022-03259-2 (PMC8991201; doi:10.1038/s42003-022-03259-2)
Supplement: Supplementary file 2 — Reporting Summary [file 42003_2022_3259_MOESM2_ESM.pdf]

nature portfolio

Corresponding author(s): Michael M. Wang

Last updated by author(s): Feb 9, 2022

Reporting Summary

Nature Portfolio wishes to improve the reproducibility of the work that we publish. This form provides structure for consistency and transparency in reporting. For further information on Nature Portfolio policies, see our Editorial Policies and the Editorial Policy Checklist.

Statistics

For all statistical analyses, confirm that the following items are present in the figure legend, table legend, main text, or Methods section.

n/a	Confirmed	
☐	☒	The exact sample size (n) for each experimental group/condition, given as a discrete number and unit of measurement
☐	☒	A statement on whether measurements were taken from distinct samples or whether the same sample was measured repeatedly
☐	☒	The statistical test(s) used AND whether they are one- or two-sided<br>*Only common tests should be described solely by name; describe more complex techniques in the Methods section.*
☒	☐	A description of all covariates tested
☒	☐	A description of any assumptions or corrections, such as tests of normality and adjustment for multiple comparisons
☐	☒	A full description of the statistical parameters including central tendency (e.g. means) or other basic estimates (e.g. regression coefficient) AND variation (e.g. standard deviation) or associated estimates of uncertainty (e.g. confidence intervals)
☐	☒	For null hypothesis testing, the test statistic (e.g. F, t, r) with confidence intervals, effect sizes, degrees of freedom and P value noted<br>*Give P values as exact values whenever suitable.*
☒	☐	For Bayesian analysis, information on the choice of priors and Markov chain Monte Carlo settings
☒	☐	For hierarchical and complex designs, identification of the appropriate level for tests and full reporting of outcomes
☒	☐	Estimates of effect sizes (e.g. Cohen's d, Pearson's r), indicating how they were calculated

Our web collection on statistics for biologists contains articles on many of the points above.

Software and code

Policy information about availability of computer code

Data collection	Proteome Discoverer (v2.4, Thermo Scientific); MassLynx v4.2
Data analysis	GraphPad Prism v.7.0c; online distribution calculator (http://www.distributome.org/V3/calc/NormalCalculator.html); MassLynx v4.2; Driftscope v3.0 (Waters Corporation)

For manuscripts utilizing custom algorithms or software that are central to the research but not yet described in published literature, software must be made available to editors and reviewers. We strongly encourage code deposition in a community repository (e.g. GitHub). See the Nature Portfolio guidelines for submitting code & software for further information.

Data

Policy information about availability of data

All manuscripts must include a data availability statement. This statement should provide the following information, where applicable:

- Accession codes, unique identifiers, or web links for publicly available datasets
- A description of any restrictions on data availability
- For clinical datasets or third party data, please ensure that the statement adheres to our policy

All data generated or analyzed during this study are included in this published article and its supplementary information files.

Field-specific reporting

Please select the one below that is the best fit for your research. If you are not sure, read the appropriate sections before making your selection.

☒ Life sciences ☐ Behavioural & social sciences ☐ Ecological, evolutionary & environmental sciences

For a reference copy of the document with all sections, see nature.com/documents/nr-reporting-summary-flat.pdf

Life sciences study design

All studies must disclose on these points even when the disclosure is negative.

Sample size	No sample size calculations were done. Sample sizes were chosen based on sample sizes used in published literature.
Data exclusions	No data was excluded from the analyses.
Replication	All attempts at replication were successful.
Randomization	This is not relevant to our study, as the study did not involve human or animal subjects.
Blinding	Blinding was not relevant to our study. For example, biochemical outcomes were quantified using automated algorithms.

Reporting for specific materials, systems and methods

We require information from authors about some types of materials, experimental systems and methods used in many studies. Here, indicate whether each material, system or method listed is relevant to your study. If you are not sure if a list item applies to your research, read the appropriate section before selecting a response.

Materials & experimental systems

n/a	Involved in the study	
☐	☒	Antibodies
☐	☒	Eukaryotic cell lines
☒	☐	Palaeontology and archaeology
☒	☐	Animals and other organisms
☒	☐	Human research participants
☒	☐	Clinical data
☒	☐	Dual use research of concern

Methods

n/a	Involved in the study	
☒	☐	ChIP-seq
☒	☐	Flow cytometry
☒	☐	MRI-based neuroimaging

Antibodies

Antibodies used

OX133 (Absolute Antibody); Anti-NOTCH3 N-terminal Fragment, UMI-D and UMI-F (Genscript); Anti-NOTCH3/N3ECD, 1E4 (Sigma Aldrich); Donkey Anti-Mouse IgG (LiCor); Anti-HA tag (Santa Cruz)

Validation

Anti-N-ethylmaleimide cysteine, OX133 is validated for use in western blotting, IP, ELISA, and FC.
Holbrook et al. OX133, a monoclonal antibody recognizing protein-bound N-ethylmaleimide for the identification of reduced disulfide bonds in proteins MAbs. 2016 May-Jun;8(4):672-7 PMID:26986548

Anti-Notch3 N-terminal fragment, UMI-D and UMI-F, are validated for use in western blotting, immunohistochemistry, and electron microscopy.
Young KZ, Lee SJ, Zhang X, Cartee NMP, Torres M, Keep SG, Gabbireddy SR, Fontana JL, Qi L, Wang MM. NOTCH3 is non-enzymatically fragmented in inherited cerebral small-vessel disease. J Biol Chem. 2020 Feb 14;295(7):1960-1972. doi: 10.1074/jbc.RA119.007724. Epub 2020 Jan 4. PMID: 31901894; PMCID: PMC7029126.

This Anti-NOTCH 3/N3ECD Antibody, clone 1E4 is validated for use in Western Blotting and Immunocytochemistry and Electron Microscopy for the detection of NOTCH 3/N3ECD.
Immunocytochemistry Analysis: A representative lot of this antibody was used to detect NOTCH 3 in CADASIL vascular smooth muscle cells (Tikka, S., et al. (2012) Journal of Cerebral Blood Flow & Metabolism. 1–10).
Immunohistochemistry Analysis: A representative lot of this antibody was used to detect NOTCH 3 in Huamn vascular smooth muscle cells & smooth muscle cells of vessels from CADASIL brain tissue (Jouet, A., et al. (2000) Journal of Clinical Investigation. 105(5):597-605).
Electron Microscopy: A representative lot of this antibody was used to detect NOTCH 3 in smooth muscle cells of vessels from CADASIL brain tissue (Jouet, A., et al. (2000) Journal of Clinical Investigation. 105(5):597-605).
Immunohistochemistry Analysis: A representative lot of this antibody was used to detect NOTCH 3 in vessels of a CADASIL patient (Rouchox, M.M., et al. (2003) American Journal of Pathology. 162(1):329-342).
Immunohistochemistry Analysis: A representative lot of this antibody was used to detect NOTCH 3 in Human colorectal carcinoma

(Serafin, V., et al., (2011) Journal of Pathology. 224(4):448-60).

HA-Tag Antibody (F-7) is validated for detection of proteins containing the HA tag by WB, IP, IF, FCM and ELISA.

Donkey Anti-Mouse IgG (IRDye 680) is validated for western blot, in cell and on cell western assays, protein array, IHC, microscopy, small animal imaging, 2D gel detection, and tissue section imaging.

Eukaryotic cell lines

Policy information about cell lines

Cell line source(s)	Human HEK293
Authentication	None of the cell lines used were authenticated, but cells were used solely for protein generation.
Mycoplasma contamination	Cell lines were not tested for mycoplasma contamination, since cells were used solely for protein generation.
Commonly misidentified lines (See ICLAC register)	*Name any commonly misidentified cell lines used in the study and provide a rationale for their use.*
